# *Calamintha nepeta* (L.) Savi and its Main Essential Oil Constituent Pulegone: Biological Activities and Chemistry

**DOI:** 10.3390/molecules22020290

**Published:** 2017-02-14

**Authors:** Mijat Božović, Rino Ragno

**Affiliations:** 1 Rome Center for Molecular Design, Department of Drug Chemistry and Technology, Sapienza University, P.le Aldo Moro 5, 00185 Rome, Italy; mijat.bozovic@uniroma1.it; 2 Alchemical Dynamics s.r.l., 00125 Rome, Italy; alchemicalduynamics.com

**Keywords:** *Calamintha nepeta* (L.) Savi, essential oil, extract, pulegone, biological activity

## Abstract

Medicinal plants play an important role in the treatment of a wide range of diseases, even if their chemical constituents are not always completely recognized. Observations on their use and efficacy significantly contribute to the disclosure of their therapeutic properties. *Calamintha nepeta* (L.) Savi is an aromatic herb with a mint-oregano flavor, used in the Mediterranean areas as a traditional medicine. It has an extensive range of biological activities, including antimicrobial, antioxidant and anti-inflammatory, as well as anti-ulcer and insecticidal properties. This study aims to review the scientific findings and research reported to date on *Calamintha nepeta* (L.) Savi that prove many of the remarkable various biological actions, effects and some uses of this species as a source of bioactive natural compounds. On the other hand, pulegone, the major chemical constituent of *Calamintha nepeta* (L.) Savi essential oil, has been reported to exhibit numerous bioactivities in cells and animals. Thus, this integrated overview also surveys and interprets the present knowledge of chemistry and analysis of this oxygenated monoterpene, as well as its beneficial bioactivities. Areas for future research are suggested.

## 1. Introduction

Essential oils (EOs) are aromatic oily liquids obtained from plant material (flowers, buds, seeds, leaves, bark, herbs, wood, fruits, and roots). They are usually complex mixtures of natural compounds, both polar and nonpolar [1], composed principally of terpenoids and their oxygenated derivatives. A variety of other molecules, such as aliphatic hydrocarbons, acids, alcohols, aldehydes, acyclic esters or lactones, and exceptionally nitrogen- and sulphur-containing compounds, coumarins and phenylpropanoid homologues, may also occur. Nowadays, EOs have received much attention as potentially useful bioactive compounds with particular emphasis on their antimicrobial, cytostatic and insecticidal activities. They have been widely used as food flavors [2], and possessing antioxidant and antimicrobial activities serve as natural additives in foods and food products [3]. Known for their antiseptic (i.e., bactericidal, virucidal and fungicidal), medicinal properties and their fragrance, they are used in embalmment, preservation of foods and as antimicrobial, analgesic, sedative, anti-inflammatory, spasmolytic and local anesthetic remedies [4].

Medicinal plants represent the basis of health care throughout the World since the earliest days of humanity and are still widely used [5]. Despite the fact that many of them have been replaced by synthetic drugs, the demand for natural products is continously increasing. Traditional use of medicinal plants encourages new phytochemical and biological screening activity, through which positive and safe therapeutic results may to be achieved. The World Health Organization (WHO) has noted that a majority of the World’s population depends on traditional medicine for its primary healthcare [6]. Many EOs and their ingredients have been shown to exhibit a range of biological activities, including antibacterial and antifungal ones. Antimicrobial properties of EOs are continuously investigated both in vitro [7,8] and in vivo [9,10] against a wide range of pathogenic bacteria and fungi. Their preparations find applications as naturally occurring antimicrobial agents in pharmacology, pharmaceutical botany, phytopathology, medical and clinical microbiology and food preservation. This review focuses on the EO of *Calamintha nepeta* (L.) Savi (CN) and one of its main chemical constituents—pulegone (PUL).

## 2. *Calamintha*: Species, Taxonomy, Occurrence and Uses

The genus *Calamintha* Mill. (calamint in English) includes aromatic plants belonging to the Lamiaceae family, which is well represented and widespread all around the Mediterranean region. Numerous members of this family are used as spices, and are also employed in folk medicine in diverse traditions. *Calamintha* species are medium to large size erect herbaceous perennials, sometimes woody at the base, represented by eight species [11] distributed in Europe, Eastern Mediterranean region, Central Asia, North Africa and America [12,13]. The number was as high as 30 before revisions in taxonomy, leaving a lot of synonyms in other Lamiaceae genera such as *Acinos* Miller, *Clinopodium* L., *Micromeria* Bentham and *Satureja* L. For this reason, the use of chemotaxonomic markers is essential to better differentiation of these genera [14]. 

The genus is represented by five polymorphic species in the European flora: CN, *C. grandiflora* (L.) Moench, *C. sylvatica* Bromf., *C. incana* (Sibth. & Sm.) Boiss and *C. cretica* (L.) Lam. [15], the last one being an endemic Cretan (Greece) species. However, according to other authors [12,13] only in Turkey there are nine species and four subspecies, with six endemic taxa, while six extremely polymorphic species are reported for the Balkan Peninsula area [16,17]. The official Italian flora [18] has recognized only three species, and *C. sandaliotica* Bacchetta & Brullo from Sardinia (Italy) has been recently described as a species new to science, morphologically related to CN [19].

Because of their pleasant mint-like smell, many *Calamintha* species are used as spices in various culinary recipes. They are used in folk medicine like mints, mainly as stimulant, digestive, tonic, antiseptic etc. [20]. These plants are used as antispasmodic, emmenagogue, diaphoretic, diuretics, carminatives expectorant and for strengthening central nervous system [13,21,22,23,24,25]. The tea is used to help with gas and colic, and externally, it is useful in poultices for bruises [20,23]. Investigations showed that leaves and flowers of *Calamintha* species are effective as an antiseptic, antispasmodic and tonic [11,17,26], as well as antimicrobial and antispasmodic activities of their EOs [11,27,28]. The oils of some species exert significant sedating and antipyretic activities in rats, likely due to the presence of the monoterpenes pulegone, menthone and eucalyptol [23,29]. *Calamintha* EOs are also used for stomach and throat aches and kidney disorders [12,13,17,30]. *Calamintha* species also have horticultural uses [21].

## 3. *Calamintha* Essential Oils

EOs are extracted from various aromatic plants, generally localized in temperate to warm areas like Mediterranean and tropical countries, where they represent an important part of the traditional pharmacopeia [4]. Recently, they have sparked interests as sources of natural products, and have been screened for their potential uses as alternative remedies for the treatment of many infectious diseases and the preservation food from either toxic effects of oxidants or from bacteria and fungi. Research on plants from different regions has led to innovative EOs uses [31]. Particularly, the antimicrobial activities of plant oils and extracts have formed the basis of many applications, including raw and processed food preservation, pharmaceuticals, alternative medicine and natural therapies [32].

The Lamiaceae family consists of approximately 200 genera of cosmopolitan distribution, many of them of economic importance due to EO production. Different *Calamintha* species vary in their EO content and composition. Moreover, the genus is characterized by highly complex chemical polymorphism, but the compounds of monoterpenoids are found to prevail, especially the oxygenated *p*-menthane-type monoterpenoids. The content of sesquiterpenoids is up to 5% [17,33].

Generally, three main chemotypes can be distinguished: PUL, piperitone oxide and carvone [34,35]. The well-known influence of origin, as well as different environmental conditions (temperature, photoperiod, nutrition and salinity) on the nature of plant chemical composition, make this complexity within the genus quite expected. The effect of different distillation methods on oil content and composition has been previously reported [36,37,38]. Other factors such as cultivation, soil and climatic conditions, harvesting time or postharvest management, can also determine EO composition and quality [39,40,41,42].

## 4. *Calamintha nepeta* (L.) Savi: Taxonomic Characterization, Distribution and Uses

**Figure 1:**
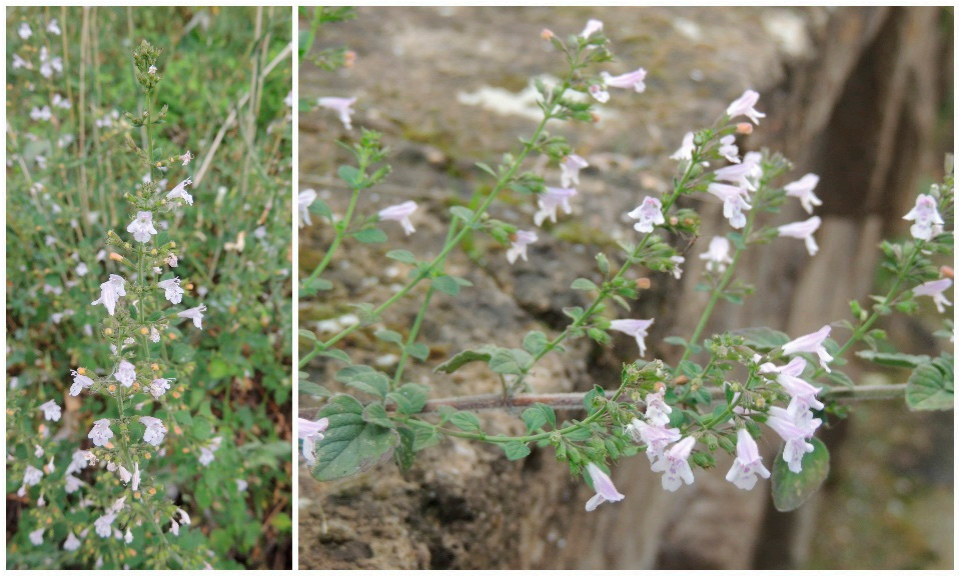
*Calamintha nepeta* (L.) Savi on its natural habitat in Tarquinia countryside (Viterbo, Italy) (photo: Mijat Božović, 2015).

Lesser calamint (CN) is a bushy, rhizomatous herb similar to the common mint in its morphology and characteristic fragrance [5,43]. It is sparsely to densely pubescent perennial up to 80 cm, with leaves broadly ovate, obtuse, subentire or shallowly to deeply crenate-serrate that are very fragrant when crushed. Leaf is with up to nine teeth on each side. It typically forms a dense foliage usually found on rocky sites, dry meadows and abandoned places. Tiny, tubular, two-lipped, lilac to white flowers appear in axillary spikes (cymes are 5- to 20-flowered). Calyx is 3–7 × 1–2 mm in diameter, sparsely to densely puberulent or pubescent, with the hairs in the mouth exserted; upper teeth are 0.5–1.5 mm in length, narrowly or broadly triangular; lower teeth 1–2 mm, puberulent, rarely with a few long cilia; corolla is from 10 to 15 mm (Figure 1). The plant grows in South, Western and South-Central Europe, northwards to East England [15,18]. Lesser calamint usually grows in late spring and summer (May/June), flowering during late summer and fruiting in autumn. Then it becomes dormant in the winter months, and re-blossoms in summer.

According to the European flora, CN includes two subspecies: *nepeta* and *glandulosa*, with the main differences in the number of flowers in cymes and leaf shape and size. Subspecies *nepeta* has 5–20 flowered cymes and leaves 20–35(–45) × 12–25(–30) mm, crenate-serrate with 5–9 teeth on each side. It is usually found on mountains of South and South-Central Europe. On the contrary, subspecies *glandulosa* has 5–11(15) flowered cymes, with leaves 10–20(–25) × 8–12 mm, subentire or shallowly crenate-serrate with up to 5 teeth on each side. It can be found in South and Western Europe [15]. These two subspecies share most of the uses and applications, since they are employed in folk medicine in diverse traditions. CN is well-known for its medicinal uses as a stimulant, tonic, antiseptic and antispasmodic [11,13,26,36,44,45]. The chemical composition of its EO has been thoroughly investigated [13,44,45,46,47,48,49,50,51,52], as well as EO antioxidant [11,17,53,54], antimicrobial [11,17,27,51,55,56,57,58,59], and anti-inflammatory activities [11,53]. In the folk medicine of different countries of the world, CN has also been widely used against insomnia, depression, convulsion and cramps [36,60] and for the treatment of respiratory and gastroenteric diseases [11,61]. An infusion of leaves is employed to treat neurovegetative distony and epilepsy [61], and its eupeptic and carminative effects improve digestion [62]. In several parts of Sicily it is used for the disinfection and cicatrization of wounds [57]. Its use against gout, cough up slime and an external application of the leaves against hip pains, has also been reported [11,63]. As a result of its antiseptic and cicatrizing activity, it is used on insect bites and wounds [64,65]. The plant has been traditionally used as a flavoring agent [18], and its EO is used in cooking as an aromatic factor and also to improve the flavor and fragrance of several pharmaceutical products [65,66]. It is also used as a spice in the Italian cuisine where it is called *mentuccia* or *nipitella*.

### Taxonomic Ambiguity and Synonyms of CN

*Calamintha* Mill., *Micromeria* Benth., *Satureja* L., *Clinopodium* L. and *Acinos* Mill. are five closely related genera of aromatic plants from the family Lamiaceae. The boundaries among them are poorly defined, and their taxonomic treatment has changed many times. Some authors treated them as only one genus, *Satureja sensu lato* [67], while others recognized *Micromeria*, *Satureja* and *Calamintha* [68,69] or five separate genera [15,16,18]. These different conclusions suggest that more characters are needed to define the generic boundaries in this plant group [14].

For this reason, the existing literature prospers in synonyms, and the genus *Calamintha* comprises different number of species depending on the authors. As already mentioned, the species in question includes two subspecies that can be found in the literature under a plenty of synonyms (Table 1).

Data collecting seems to be inexhaustible, and even bigger problem arises when it comes to the level of subspecies. Furthermore, sometimes the plant material was not characterized in the terms of subspecies (but determined only to the level of species), which makes it particularly hard to draw conclusions and to compare the existing data. Thus, their separation seems to be irrelevant, especially taking into account the possibility of uncompleted or even incorrect determination of material. On the other side, the question of valid recognition of these two taxa has been the subject of some earlier studies. Three population of CN have been investigated from a morpho-anatomical and phytochemical points of view. According to the authors it is impossible to distinguish the two taxa in CN complex on morphological grounds [70]. Morever, the forms at opposite ends of the range of morphological variations can be displayed by individuals of a single population at different stages of their life cycle and at different times of the year. In addition, a number of phytochemical studies have been performed, particularly on the EOs and volatile components. These studies have sometimes attempted to contribute to a taxonomic understanding of the group, at generic [71], specific or intraspecific level [72,73]. However, according to the main part of literature, it can be concluded that the chemical composition is quite independent of the subspecies and that they both can produce the same volatiles with the *p*-menthane skeleton oxygenated in C-3 [34,44,46,47,48].

The nomenclature situation of these two taxa has been thoroughly discussed [74]. Examination of the diagnostic characteristics of the two subspecies has shown that many of them overlap, and together with evidence from phytochemistry, led to the conclusion that it was impossible to find any features which would allow the two taxa to be distinguished in practice. According to them, these are the seasonal morphs rather than discrete taxa, and they not deserve recognition at any rate higher than a super-varietal level.

Taking everything mentioned into account, hereby we have indiscriminately collected data of either subspecies, as well as of the numerous synonyms, marking them all as CN (the level of species).

## 5. Essential Oil Composition of CN

The literature data clearly indicate the presence of a remarkable chemical polymorphism and great intraspecific variability. Some authors observed that the oil composition seemed to be independent of the geographical origin of the sample [34,75], while others reported a strong chemical variability depending on the origin of the samples [51], as well as environmental conditions on the nature of plant chemical composition [17]. Described chemotypes are diverse, but the major components in the oils generally belong to the C-3 oxygenated *p*-menthanes such as PUL, menthone, isomenthone, and piperitone and piperitenone with their oxides or, more rarely, C-6 oxygenated *p*-menthane compounds such as carvone (Table 2) [17,23]. Our literature survey has discovered plenty of data, but at least three types of oils can be distinguished, with some exceptions:

(I) The first and the most abundant one consists of PUL as the major component associated with other compounds. Two main variants of this chemotype can be defined as follows: (1) the one presented by PUL and menthone and/or isomenthone, menthol and its isomers, and (2) the other where PUL is associated with piperitenone, or piperitone and piperitenone oxides;
(II) The second chemotype can be considered as piperitone oxide or the piperitone oxide/piperitenone oxide one. A piperitone/piperitenone variant of this chemotype was only reported for some Croatian material [17];
(III) Lastly, the rarest one is distinguished by the presence of carvone and 1,8-cineole.

Due to the difficulty to summarize all the data available, here they are reported as groups distinguished by the geographical origin of the material used in the analysis.

Italian material from different regions has been investigated extensively and plenty of data are available in the literature. All three chemotypes have been reported. About 50% of PUL in the oil from Tuscany was reported, and menthone (9.4%), limonene (7.0%), menthol (4.6%), piperitenone-oxide (4.6%) and piperitone-oxide (3.9%) were also present [57]. Samples from Sardinia were characterized by the high content of PUL (39.9%–64.4%), but piperitenone (6.4%–7.7%) and piperitenone oxide (2.5%–19.1%) were also found to be important components [51]. A rather big amount of PUL was reported for the stems and cymes (30% and 28%–52%, respectively) collected in Pisa area [70], and the same analysis included stem samples rich in piperitone oxide (13%–38%), and both stems and cymes with the high content of piperitenone oxide (18% and 22%, respectively). Additional study included material from this region showing PUL (46%), menthone (9.82%), limonene (6.4%) and menthol (4.82%) as major components [55]. The samples from Sicily are also characterized by PUL (21.4%–25.2%) and other ketones with *p*-menthane skeleton, such as menthone (11.6%–19.8%), piperitone (6.4%–13.1%), piperitenone (12.3%–16.4%) and isomenthone (2.1%–12.0%) [36]. Another analysis on the material collected in Urbino [73] showed the particular richness in piperitone oxide (68%) with the presence of a limited amount of piperitenone oxide (3.4%), as well as the presence in significant quantities of limonene (2%), isopulegol (4%), isopulegyl acetate (2.3%) and thymol (1.2%). A comprehensive study on different samples from Tarquinia countryside was also recently reported [76]. The material was analyzed in terms of different harvesting period (4 months, from July to October 2014) and extraction duration (from 1 to 24 h). The analysis revealed the PUL/menthone chemotype, but the ratio varied greatly according to the plant’s phenophase, with the greatest increase of menthone percentage during the fruiting period. PUL was particularly abundant in July and August (77.7% and 84.7%, respectively), reaching its maximum during the first three h of extraction, while menthone percentage increased at the blooming period (up to 35.4%). Chrysanthenone gradually increased its amount with the extraction time (up to 33.9%), and a significant amount of limonene was observed (up to 13.6%). Leaves, stems and flowers were sampled in Calabria at the beginning of the flowering stage, and PUL was the main constituent (58.85%), along with *p*-menthone (7.45%), menthol (3.89%) and piperitenone (3.34%) [77]. Another study included the material from Apulia and reported the presence of four oil types: piperitone oxide, piperitenone oxide, piperitone-menthone and PUL [52]. The material from Liguria region (North of Italy) was characterized by 23.6% of PUL, 15.8% of piperitenone, 15.3% menthone and 14.9% of piperitenone oxide among others [56]. The material sampled in Piedmont region at the full bloom showed the predominance of PUL (73.65%) and the lack of menthone (only 0.25%). *cis*-Piperitone (4.56%), limonene (3.54%), *cis*-piperitone oxide (2.48%), isomenthone (2.39%), germacrene D (2.35%) and isopulegone (1.89%) were also found [42]. The work included different postharvest management (dehumidifying and oven-drying of the material) impacts on the yield and the EO chemical profile. They both were significantly affected, with the dehumidifying process leading to a significantly higher EO content than the oven-drying process (2.625% and 0.101%, respectively, in regard to 0.242% in the fresh material). PUL was not significantly influenced by the treatments. The amount of limonene was decreased by the both processes (up to 36.2% less), while the sesquiterpenes germacrene D and β-caryophyllene were affected mainly by the oven-drying method (86.5 and 157.3% higher than the fresh samples). The same method greatly increased the menthone content (total of 5.38% in the oil). Additionally, some analysis on the material of Italian origin differ largely, supporting the hypothesis of the great chemical variability present in this species. For instance, the oil from the Campania region was characterized by a prevalence of the sesquiterpenic fraction, with 1,10-di-*epi*-cubenol (18.5%), cadalene (5.7%) and *allo*-aromadendrene epoxide (11.4%) as the main constituents [24], while the high content of carvone, followed by carvacrol and limonene, has also been reported for the material from different areas of the “*Appennino marchigiano*” [78]. High contents of carvone (64.3%) and PUL (10.9%) in the oil obtained from a commercial source have also been reported [79].

An important chemical variability has been observed for CN growing wild in Corsica [34]. A total of 40 samples was analyzed (Table 3), and the half was characterized by the predominance of menthone (mean content 43.4%). PUL (18.9%), *trans*-piperitone oxide (8.3%) and limonene (5.2%) were also important constituents. Other compounds occurred occasionally in substantial amounts: isomenthone (up to 16%), piperitone (up to 12.2%), neomenthol (up to 10%) and piperitenone (up to 7.1%). Thirteen samples from that study were characterized by a strong predominance of PUL (55.6%) over menthone (20%), and all the samples contained regular amounts of limonene (6%). *trans*-Piperitone oxide (30.5%) and piperitenone oxide (12.5%) were the main components of the last 11 samples, with the large amounts of limonene (12.8%), while PUL and menthone were less represented. According to the authors, PUL is strongly predominant from the beginning of the vegetative life to August (63%–78%). Its percentage reaches a maximum in early July and decreases at the beginning of the flowering stage, while the amount of menthone undergoes the reverse evolution. It seems possible to establish a negative correlation between these two compounds [34]. The other analysis included material from the same area, showing PUL/menthone chemotype as the most frequent one. These two compounds represented together between 70% and 85% of the total oil composition, but the ratio varied greatly in terms of growth plant stage: the menthone percentage increased at the blossoming period, while PUL% decreased [44]. This is in accordance with another study of the material harvested in autumn in the vicinity of Marseilles, showing menthone (52.7%) as the most dominant compound, along with PUL (9.1%), piperitone (7.8%), neomenthol (7.6%), menthol (4.3%) and limonene (4%) [80].

The material from Greece has also been found to have very variable composition. One study reported 39.7% of PUL in the oil from the island of Lefkada, with menthone (24.7%) and isomenthone (25.6%) among others [49]. The presence of at least 46 compounds in the oil was also reported, and the major ones were found to be the two diastereoisomers of piperitone oxide (55%) and β-bisabolene (8.5%) [81]. Another study on material of Greek origin showed 41% of PUL and 32% of menthone, with piperitone (7.3%) and piperitenone (7%) as important constituents [82], while two chemotypes were noted by others [75]. The first was rich in PUL and/or menthone and/or isomenthone, and the sum of the contents of these three ketones ranged from 56.8% to 89.9%. The second was found to be rich in *cis*- and *trans*-piperitone oxide and/or piperitenone oxide, and the sum of these three epoxides ranged from 65.5% to 90%. The co-occurrence of two chemotypes within the same population (intra-populational variation) has also been reported for the material on the Island of Zakynthos [35]. C-3 oxygenated *p*-menthane compounds and their precursor limonene constituted from 68.8% to 92.8% of the oils. The main constituents of the first chemotype were PUL, menthone, piperitenone and piperitone, while *cis*- and *trans*-piperitone oxide, limonene and piperitenone oxide were noted for the second one. The study included different plant organs (inflorescences, leaves and stems) and different development periods of plant (vegetative and flowering stages). Differences in the percentage of the main constituents were observed between the oils from the different organs in the same developmental stage, as well as between the same organs in different developmental stages.

Several studies on the Turkish material confirmed the presence of different chemotypes. 23 components have been identified, with PUL (42%) and piperitenone (40.4%) being the major ones [50]. Another analysis of the Turkish material showed PUL and menthone (40.5% and 23.6%, respectively) as the more important components, followed by piperitone and piperitone oxide (9.3%) [45]. Oxygenated monoterpenes PUL (54%) and menthone (16%) were also found to be the major constituents of the oil from Istanbul [11], while the predominance of piperitenone oxide (43.8%), *trans*-piperitone oxide (25.2%) and limonene (13%) was reported by other authors [83]. A comprehensive study on the samples from different regions in Turkey (Icęl, Bartin, Zonguldak and Manisa) was reported [13]. The analysis confirmed the dominance of 3-oxo compounds, but also showed that their 1,2-epoxy derivatives may predominate in some oils. *trans*-Piperitone oxide (44.4%), piperitenone oxide (11.7%) and limonene (7.1%) were the main constituents in some samples, while other had *trans*-piperitone oxide (30.9%) associated with caryophyllene oxide (7.8%). The high presence of PUL (up to 19.5%) and menthone (up to 11.9%) was noted for some samples, but carvacrol (10%) and limonene (7.5%) were also found in the part of them. A type containing caryophyllene oxide (7.9%), *trans*-piperitone oxide (5.7%) and menthol (5%) was also detected. Others [84] have confirmed the prevalence of *cis*-piperitone epoxide (48.66%), piperitenone oxide (22.08%), limonene (13.51%) and terpinen-4-ol (4.55%), while other analysis of the Turkish material showed PUL (76.5%) and piperitone (6.1%) as the main constituents [85]. *cis*-Piperitone epoxide (48.66%), piperitenone oxide (22.08%), limonene (13.51%) and terpinen-4-ol (4.55%) have been also reported as the main compounds [60], and the authors highlighted prevalence of monoterpenes (89.19%). The oils obtained from wild plants growing in Mediterranean region of Turkey revealed two different oil’s composition: one rich in piperitone oxide (33.78%), piperitenone oxide (15.79%) and isomenthone (11.17%), and the other with PUL (48.44%) and menthone (38.69%) in prevalence [86].

The oil extracted from Portuguese material was predominantly composed of isomenthone (35.8%–51.3%), 1,8-cineole (21.1%–21.4%) and *trans*-isopulegone (7.8%–6.0%), which makes this oil quite peculiar and rather different from those of other origins; the authors marked it as a new oil type [51].

The presence of carvone (37.6%) and 1,8-cineole (34.9%) along with limonene (11.5%) as the main compounds has been reported for the Spanish material presenting the 2-oxygenated *p*-menthanic type [87]. According to the authors, a simple dominant gene A favors production of PUL from piperitone, but its lack leads instead to the production of terminal types, such as piperitone, 1,2-epoxy-*p*-menthanes or menthone/isomenthone. Carvone production appears to be due to a single dominant gene C, which is epistatic for the gene involved in the complex system of 3-oxygenated *p*-menthane constituents; this gene is apparently linked to another dominant gene Lm which leads to limonene production. 

Detailed chemotaxonomic studies were undertaken on the Belgian material [46,47,48]. The oils’ composition ranged from the very simple, containing almost exclusively piperitone oxide and piperitenone oxide (30.7%–74.2% and 4.7%–52%, respectively), to the relative complex, containing the oxidized precursors PUL, menthone, menthol and their acetates. The authors noticed that the high concentrations of piperitone oxide and piperitenone oxide were found to be depended on the maturity of the plants [46]. On the other hand, several samples they analyzed contained considerable amounts of PUL (up to 57.7%), menthol and menthone, but were also rich in the oxides of piperitone and piperitenone [48].

A Montenegran sample was rich in PUL (37.5%), menthone (17.6%), piperitenone (15%) and piperitone (10.2%) [58], while the predominance of carvacrol (79.91%) was reported by others [88].

The min component (approximately 37%–45%) of the oils from material naturally occurring in Croatia varied as follows: PUL (three samples), piperitenone oxide (two samples) and menthone (one sample) [89]. For this region, the prevalence of oxygenated monoterpenes with piperitone (19.9%–59.5%) and piperitenone (7.1%–42.6%) as the main representatives has also been reported [17], while the material from Dalmatia, near Split, has been found to contain monoterpene oxides such as piperitone oxide (46.0%), piperitenone oxide (12.7%) and limonene (10.9%) [90].

The material from Serbia was rich in PUL (75.5%), piperitenone oxide (6%), menthone (5.3%) and menthol (4.3%) [33], while another study showed the predominance of piperitone oxide (59.09%), with limonene (9.05%), *cis*-sabinene hydrate (4.45%) and 4-terpineol (4.77%) among others [59].

A high amount of easily volatile compounds was remarked in the oils extracted from the material cultivated in Romania [25]. Some samples were characterized by a great content of PUL (48.7%) followed by *p*-menthone (28.3%), while others contained estragol as the major compound (54.95%), followed by mentone (20.19%) and PUL (12.87%).

Carvone (46.7%), PUL (22.1%) and limonene (24.6%) as the main components were found in the material from Iran [91]. Other authors [92] showed that sesquiterpene hydrocarbons (β-bisabolene 9.9%, germacrene D 7.6%, β-bourbonene 7.4%) and monoterpene ketone piperitone (5.3%) are the major compounds of the oil with the same origin.

Analysis of Egyptian material revealed 64 components with carvone as the main one (38.7%), followed by *neo*-dihydrocarveol (9.9%), dihydrocarveol (6.9%), dihydrocarveol acetate (7.6%), 1,8-cineole (6.4%), *cis*-carvyl acetate (6.1%), and PUL (4.1%) [65]. Others [93] have reported 1,8-cineole (36.6%) as the major component, followed by PUL (17.9%) and limonene (9.2%) for the Moroccan material, while other analysis of the same origin material found *ρ*-cymene (20.9%), *γ*-terpinene (18.7%) and thymol (34.94%) as the most abundant constituents [94]. The material sampled in North Morocco gave the oil rich in 1,8-cineole (42.94%), β-phellandrene (11.39%) and pinocamphone (9.88%), with a high proportion of monoterpenes (76.09%) in comparison with sesquiterpenes (7.29%) [95]. Aerial parts at the flowering stage were sampled in Algeria, and the most abundant components of the oil were menthone (26.46%), piperitone oxide (22.26%) and PUL (14.04%) [96]. Another study from this region showed the oxygenated monoterpenes as the predominant class, with PUL (39.5%), *neo*-menthol (33%) and isomenthone (19.6%) as the major constituents [97].

As a part of the research on aromatic plants from Argentina, the oil composition of the leaves of CN has been examined showing PUL (34.28%), neomenthol (30.61%) and menthone (17.12%) as major constituents [98].

The oil from a cultivated population of CN in South India revealed the prevalence of menthone (43.4%), with significantly lower amounts of piperitone oxide, carvone, menthol, piperitenone, limonene, linalool and *beta*-caryophyllene [99]. The author marked it as a new menthone chemotype.

## 6. Antimicrobial Activity of CN

Infectious diseases represent an important cause of morbidity and mortality among the general population, particularly in developing countries [100]. However, due to indiscriminate use of antimicrobial synthetic chemicals in their treatment, both human and plant pathogenic microorganisms have developed resistance to multiple drugs/chemical substances. In addition, these chemical compounds can cause undesirable effects on environment because of their slow biodegradation and serious side effects on mammalian health because of toxic residues in agricultural products [59,84,101,102]. Consequently, there is an increased interest in developing new antimicrobial drugs from various natural sources, especially aromatic and medicinal plants. 

Among many plant products, EOs are the most studied plant secondary metabolites [103]. Along with extracts, they represent potential sources of novel antimicrobial compounds. Their antimicrobial evaluations are generally difficult because of their volatility, insolubility in water and complex chemistry [104,105]. However, the antimicrobial activity of EOs has been extensively studied and demonstrated against a number of microorganisms, usually using direct-contact antimicrobial assays, such as different types of diffusion or dilution methods, as reviewed by many authors [2]. In these tests, EOs are brought into direct contact with the selected microorganisms. Due to their high hydrophobicity and volatility, the direct-contact assays face many problems. In diffusion assays, the EOs components are partitioned through the agar according to their affinity with water, and in dilution methods low water solubility has to be overcome by addition of emulsifiers or solvents (such as DMSO or ethanol) which may alter the activity [106]. 

A wide variety of EOs is known to possess antimicrobial properties due to the presence of active monoterpene constituents [4]. Antimicrobial action is often determined by more than one component; each of them contributes to the beneficial or adverse effects. The major component may not be the only one responsible for the antimicrobial activity but a synergistic effect may take place with other oil components [2,107]. Possible interactions among conventional drugs and products obtained from medicinal plants are observed, motivating researchers to test the possibilities of their synergism. It must be emphasized that these interactions depend on several factors including pharmacokinetics and employed doses, since combinations confirmed in vitro may not have the same effect on humans [100,108].

According to the literature survey, different calamint species have been investigated in search for antimicrobial activities [79,95,109,110,111,112], including several analyses performed on CN with an aim to investigate its antibacterial, antifungal or antiviral effects.

For instance, the oil from the Algerian material was assayed for its antimicrobial activity against six bacteria and two fungi, resulting in a range of growth inhibition patterns against pathogenic microorganisms (MIC values from 0.125% to 0.5%). The results revealed *Staphylococcus aureus* as the most sensitive bacteria (the MIC value was 0.25% and the inhibition zone was 40.6 mm), while *Pseudomonas aeruginosa* appeared as the most resistant one [97]. The authors noted that the antimicrobial activity could be attributed to its high content of compounds with known antimicrobial activity, such as menthone and PUL. Another analysis on the same origin material assessed antifungal activity against two post-harvest pathogenic fungal strains from *Fusarium* and *Aspergillus* genera [96]. The results in vitro demonstrated an excellent antifungal property of this oil, and the authors pointed out that this activity can be attributed to oil’s high amount of menthone, piperitone oxide and PUL. A group of authors from Algeria investigated the effectiveness of increasing doses of three EOs (including CN) in the inhibition of methanogenesis in the rumen, and their effects on in vitro ruminal fermentation traits of vetch-oat hay [113]. Addition of EOs in the culture media at all doses reduced methane production, with the effect more pronounced after 24h of incubation. As reported, this was mainly attributed to antimicrobial activity of EOs since they exert antimicrobial activity by multiple mechanisms of action and can inhibit a broad variety of both Gram(+) and Gram(−) bacteria and other ruminal microorganisms [114,115,116]. The results also showed the decrease of ammonia N concentrations and some authors suggest that EOs reduce ammonia N by inhibition of deamination which is assured principally by ammonia hyperproducing bacteria [117]. According to some authors [118], the decrease in ammonia concentration could result from a negative effect on protozoa and thus a decrease in predation of bacteria. 

The oil distilled from Moroccan plants failed to show antifungal activity against *Botrytis cinerea* [93], while another study included five Gram(+) and four Gram(−) bacteria [95]. Depending on the microorganism tested, the last analysis showed from good to moderate activity of EOCN, with the MIC value from 0.5 to 14 µL·mL^−1^. The most susceptible bacterium was *Listeria monocytogenes*, while *Salmonella Senftenberg* and *Yersinia enterocolitica* seemed to be the most resistant ones. Quite good activity was also noticed against *S. aureus* (2 µL·mL^−1^) and *Bacillus subtilis* (4 µL·mL^−1^). Regarding the bactericidal activity, Gram(+) bacteria were more susceptible than Gram(+) ones, and no bactericidal effect was observed against *S. Senftenberg*, *Y. enterocolitica* and *Enterococcus faecium.* In general, when EOs act alone, their high doses are required to achieve the significant antimicrobial effect. Therefore, the development of new strategies that allow the increase of their efficacy at lower concentrations is needed. The authors have also analysed a possible synergistic lethal effects against *Escherichia coli* and *L. monocytogenes* in combination with mild heat or emerging methods: high hydrostatic pressure (HPP) and pulsed electric fields (PEF). The results demonstrated the occurrence of additive and outstanding synergistic lethal effects when combining all three treatments with the EO at the low dose proposed (0.2 μL·mL^−1^). The authors suggested sublethal injuries caused by the heat, making cells sensitive to the bactericide action of the oil. According to them, that could be related to the high content of PUL and 1,8-cineole. Regarding the HHP treatment, the outstanding synergistic effect was observed, especially against *L. monocytogenes*, whereas the combination with PEF treatment was much less effective showing additive effect rather than synergistic. Therefore, the obtained data confirmed the possibility of the application of alternatives to traditional treatments, offering a great potential by reducing treatment intensity and doses of antimicrobials, and consequently adverse effects on food quality, while enhancing the antimicrobial security of foods [119,120,121,122]. 

Egyptian material rich in carvone [65] showed a significant antimicrobial activity, and the strongest one on *S. aureus*, *Candida albicans* and *Aspergillus niger*. The authors highlighted carvone’s antimicrobial activity reported by some authors [123,124], as well as its possible synergistic effect with minor components that possess antimicrobial activity [27,125,126].

Evaluation of Minimal Inhibitory Concentration (MIC) and Minimal Lethal Concentration (MLC) values included two samples from Sardinia and Portugal [51]. The oils were tested against different *Candida* strains (*C. albicans*, *C. tropicalis*, *C. krusei*, *C. guillermondii* and *C. parapsilosis*) and the following dermatophytes: *Trichophyton rubrum*, *T. mentagrophytes*, *Microsporum canis*, *M. gypseum, Cryptococcus neoformans*, *Epidermophyton floccosum*, *A. niger*, *A. fumigatus* and *A. flavus*. The Italian oil was more active than the Portuguese one, exhibiting the significant antifungal activity against *Aspergillus* and dermatophyte strains, with MIC values from 0.32 to 1.25 µL∙mL^−1^. The authors concluded that the highest antifungal activity of the Sardinian oil can be associated with the contribution of PUL, and suggested its use for therapeutical purposes, particularly in the treatment of dermatophytosis and aspergillosis.

The antimicrobial investigations of Italian material collected in Pisa area [57] were performed against the Gram(+) bacteria *L. monocytogenes* and *Bacillus cereus*, the Gram(+) *Salmonella veneziana*, *S. paratyphi B* and *S. typhimurium*, and the fungi *Fusarium moniliforme*, *Botrytis cinerea*, *Aspergillus niger* and *Pyricularia oryzae*. The oil showed a wide antimicrobial spectrum of action, and noteworthy is the effectiveness on some mycetes parasites of higher plants, as well as the strong activity against all the *Salmonella* species, etiologic agents of many food poisonings. Some authors have marked the oil from this region as a very powerful one [55]. In that study, bacteria *S. aureus*, *E. coli*, *P. aeruginosa* and *B. subtilis* were tested, as well as fungi *Saccharomyces cerevisiae* and *C. albicans*. The PUL-rich oil showed good potency against all tested microorganisms, particularly against *B. subtilis* (MIC was 2 µg∙mL^−1^). The authors emphasized as an interesting fact that *P. aeruginosa*, known to be very resistant even to synthetic drugs, was found to be very susceptible to the oil. According to the authors, the activity could be due to the presence of the ketones menthone and PUL or piperitone and piperitenone with their oxides. The fungistatic and fungicidal activities of the oil from Italian material, even at low doses, were detected in vitro on *M. canis* and *M. gypseum*, mycetes responsible for human cutaneous mycoses spread by domestic animals [56].

The in vitro anti-*Candida albicans* activity of 44 samples from Tarquinia has been reported recently [76]. The samples were prepared from the material collected in four different months and by steam distillation process of different duration (1 to 24 h). The oils were found to belong to the PUL-rich chemotype, with the particular increase of menthone amount during the reproductive periods of the plant (September-October). With few exceptions, the MIC of this strain ranged from 6.24 mg∙mL^−1^ to 12.48 mg∙mL^−1^ for the oils extracted in July and October, and from 3.12 to 12.48 mg∙mL^−1^ for the ones extracted in August and September. Notably, some samples showed an interesting and significant antifungal activity with MIC ranging from 0.78 to 1.56 mg∙mL^−1^. Usually, the third fraction (between second and third h of extraction) showed good activity, or even the fourth one (between third and sixth h) in the case of September. October was characterized by lack of any significant activity. These results are not in accordance with the observations indicating that PUL and/or menthone are the constituents responsible for the antimicrobial activity of EOCN. It is clear that other minor chemicals are endowed with microbiological activities and may co-participate in the inhibition process with some synergistic mechanism [107] and it is somehow in agreement with the phytocomplex hypothesis reported in many other experimental observations [127,128]. The analysis has also indicated the lack of any significant correlation between the antimicrobial activity and the plant’s phenophase. Somehow, this is in contrast with some bibliographic data reporting that EOs obtained during flowering season of a plant exhibit the most significant antimicrobial activity [2,129,130].

The oil from Montenegro was screened against the bacteria *E. coli*, *S. aureus*, *B. subtilis*, *P. aeruginosa, Salmonella enteritidis* and the fungus *A. niger*. All the microorganisms except *S. enteritidis* were found to be susceptible, specifically *A. niger* [58]. This was confirmed by another study which included the material collected in Serbia and the same test microorganisms [33]. It was found that all microorganisms were susceptible to the oil at all oil dilutions; however, the oil activity declined with dilution. The material from Serbia was tested against 11 model bacteria, and the results showed that the EO possessed antimicrobial activity against all tested microorganisms with the range of MIC values from 0.025 to 1.56 µL·mL^−1^ and MBC (Minimal Bactericidal Concentration) values from 0.05 to 1.56 µL·mL^−1^ [59]. The low activity was observed against Gram(−) *S. enteritidis* and *E. coli* (0.78 µL·mL^−1^) and Gram(+) strains of *S. aureus* (1.56 µL·mL^−1^), while *B. cereus* and *P. aeruginosa* were moderately sensitive to the oil (0.1 µL·mL^−1^). The highest activity (0.025 µL·mL^−1^) was found with some ATCC strains of *P. aeruginosa* and *B. cereus*, as well as against *E. coli* from feces.

Antimicrobial activity of the Turkish sample was evaluated, using the disc diffusion method. That study showed that all the tested bacteria (particularly *B. subtilis*, *Staphylococcus epidermidis*, *Stenotrophomonas maltophilia*) and *C. albicans* were affected by the EO. The authors explained that by the high percentage of PUL and menthol [27]. The antibacterial properties of the oil obtained from the Turkish material was evaluated against 20 phytopathogenic bacteria [84]. The results revealed that the oil exhibited strong antibacterial activities against most of the tested bacteria. Both Gram(+) and Gram(−) bacteria were sensitive (the MIC value was 7.81 μg·mL^−1^), with no significant difference in susceptibility between them. The analysis included the following microorganisms: *Clavibacter michiganensis*, *Bacillus pumilus*, *Enterobacter intermedius*, *Erwinia caratovora*, *E. chrysanthemi*, *Pseudomonas fluorescens*, *P. cichorii*, *P. corrugate*, *P. syringae (pv. syringae* from different hosts, *pv. tomato*, *pv. phaseolicola*, *pv. pisi* and *pv. tabaci)*, *Agrobacterium tumefaciens*, *Ralstonia solanacearum*, *Xanthomonas vesicatoria* and *X. axonopodis pv. campestris*. The authors associated the high antibacterial effect with the presence of many components, pointing out the possible synergistic and antagonistic effects of the main chemicals and minor components in the oil.

However, assessing EOs food protection, CN was tested against 10 bacteria and failed to show any significant efficacy [131].

The mechanisms by which EOs inhibit microorganisms involves different modes of action, but may be due in part to their hydrophobicity. As a result, they cause lipid partitioning of bacterial cell membranes and mitochondria, disturbing the cell structures and rendering them more permeable [100,132,133,134]. Extensive leakage from bacterial cells or the exit of critical molecules and ions, will lead to death [6]. Impairment of bacterial enzyme systems may also be a potential mechanism of antimicrobial action [135]. Cyclic hydrocarbons act on ATPases, enzymes known to be located at the cytoplasmic membrane and surrounded by lipid molecules. In addition, lipid hydrocarbons may distort the lipid-protein interaction, and the direct interaction of lipophilic compounds with hydrophobic parts of the protein is also possible [100,134]. It was found that some EOs stimulate the growth of pseudo-mycelia, indicating that they may act on enzymes involved in the synthesis of bacterium structural components [136].

The antimicrobial activity of the EOs can also be explained by the lipophilic character of the monoterpenes contained. The monoterpenes act by disrupting the microbial cytoplasmic membrane, which thus loses its high impermeability for protons and bigger ions. If the membrane integrity is disrupted, then its functions are compromised not only as a barrier but also as a matrix for enzymes and as an energy transducer. However, specific mechanisms involved in the antimicrobial action of monoterpenes remain poorly characterized [137]. According to a number of authors, Gram(−) bacteria are generally less susceptible than Gram(+) bacteria to the actions of EOs, due to their outer membrane surrounding the cell wall which restricts diffusion of hydrophobic compounds through its polysaccharide covering [138,139,140,141]. This effect seems to be dependent on lipid composition and net surface charge of microbial membranes [139]. This statement is not always true; indeed different authors found no differences or greater sensibility of Gram(−) bacteria than Gram(+) to EOs [142,143].

Some antimicrobial and antifungal abilities have been reported for monoterpenes [144], and some *Calamintha* species have been investigated as well [71]. The authors concluded that the activity appeared to be due to the monoterpene compounds of the oil [71]. It is also known that *p*-menthane ketones are effective towards a number of microorganisms [145], while PUL has found to exhibit high antimicrobial activity, especially on *S. typhimurium* and *S. aureus* [146]. Also, it was demonstrated that PUL is the constituent responsible for the antimicrobial activity in the oil of CN [57].

In addition, different extracts and fractions were investigated against Gram(+) *B. subtilis* and Gram(−) *E. coli* and *S. typhimurium*. The analysis showed better activity of the water extract then the methanolic one, while the fractions prepared had greater bactericidal efficacy, especially the dichloromethane one. However, methanolic extract and the ethyl acetate fraction failed to inhibit all tested bacteria [147]. Another study assessed the antimicrobial activities of the methanolic, ethanolic and petroleum ether extracts of CN of Turkish origin, using the disc diffusion and microdilution methods. As a result, strong antifungal effect was found just for the ethanolic and petroleum ether extracts against *Fusarium proliferatum* (1.6 mg∙mL^−1^), while the other tested bacteria and fungi were inhibited only by the higher concentrations (from 6.3 mg∙mL^−1^ to 12.5 mg∙mL^−1^) [148]. Methanolic extract from the Turkish material from Sinop showed good antimicrobial effect against *E. faecalis* and *C. parapilosis* [149].

In addition, the preservative activity and potential of CN and its use in cosmetics have been also presented [79,150]. Preservatives are included in pharmaceutical and cosmetic formulations to protect the product from microbial insults occurring from raw materials, manufacture and consumer use. A recent trend in cosmetic preservation is to avoid the use of chemical agents, leading scientists to search for natural antimicrobial alternatives. EOCN from a commercial source was analyzed and showed antimicrobial activity against *E. coli*, *S. aureus*, *C. albicans* and *A. niger* [79]. On the contrary, its activity against *P. aeruginosa*, an organism intrinsically resistant to a wide variety of antimicrobial agents, was unsatisfactory. The authors hypothesized that it was probably due to the impermeability of the outer membrane to hydrophobic and high molecular weight hydrophilic drugs, as it has been already reported [151,152]. However, the good activity of the association EO–EDTA against *P. aeruginosa* confirms that while the essence itself hardly crosses the outer membrane of the bacterium, its combination with EDTA allows it to reach the inner part of the cells. In fact EDTA chelates the Ca^2+^ and Mg^2+^ ions that play an important role in the stability of the outer membrane complex, as it is reported [153]. The preserving activity of the EOCN in cetomacrogol cream was proved to be satisfying, although less than one observed in culture medium. According to the authors, effective preservation was also achieved thanks to the high concentration of carvone which was a main constituent of EOCN.

In the other study, the oil was assayed for its activity in two different formulations (cream and shampoo). Preservation effect was limited to higher concentration (2%), with no significant differences between standard and wild strains either in single or mixed cultures, but the nature of formulation in which it was incorporated had considerable effect on its efficacy [150]. The higher concentration required for good preservation of the cream, could be explained by the lipophilic affinity of the EO for liquid paraffin, the major cream’s compound. Although EOs are often highly lipophilic compounds insoluble in the aqueous phases, in many cases they have a relative hydrophilicity given by the presence of constituents with polar functional groups. According to the authors, that oil’s affinity for liquid paraffin probably reduced the EO bioavailability in aqueous phase, requiring higher percentage to avoid bacterial recovery and/or possible infection spreading. This was confirmed by some other authors [154]. In contrast, the antimicrobial activity was more effective against the Gram(−) bacteria that may have been due to the presence of EDTA in the cream that acted as synergic agent. In the case of shampoo formulation, used concentration was insufficient to reduce the Gram(+) inoculum. The authors explained it by the presence of surfactants, already reported in the literature [154,155]. On the contrary, for Gram(−) bacteria, the EO-preserved shampoo showed a satisfactory preservative efficacy. Also here, interfering factors could have influenced the oil effectiveness. Surfactants are organic compounds composed of both hydrophobic and hydrophilic parts, normally found in detergents and shampoos. They reduce surface tension, thereby diminishing interfacial forces and leading to the formation of a micellar structure. The affinity of the EO for the surfactant micelle could have lowered the oil bioavailability and then partially neutralized its antimicrobial activity as well as liquid paraffin in the cream formulation [150].

## 7. Antioxidant Properties of EOCN

Free radicals are considered to initiate oxidation that leads to aging and causes diseases in human beings. Moreover, activated oxygen incorporates reactive oxygen species (ROS) which consists of free (hydroxyl or superoxide anion radicals) or non-free radicals (peroxide) [156]. ROS are liberated by virtue of stress, and thus, an imbalance is developed in the body that damages cells in it and causes health problems [157]. On the other hand, restriction on the use of synthetic antioxidants has been imposed, because of their carcinogenicity and other toxic properties, which has increased considerably the interest in natural antioxidants [31,158].

The active ingredients of a medicinal plant are mainly its secondary metabolites, and natural antioxidants can be phenolic compounds (tocopherols, flavonoids and phenolic acids) or carotenoids (lutein, lycopene and carotene) [31,159]. The interest in phenolic antioxidants has increased remarkably in the last decade due to their great capacity to scavenge free radicals associated with various human diseases. Phenolic compounds, particularly flavonoids which are present in all vascular plants, are involved in many physiological processes; they are stress biomarkers ensuring the survival of plants under different environmental conditions, but they are also included in plant protection. In general, the antioxidative effectiveness of plant extracts depends of the content of phenolic compounds and the reaction activity of the phenol towards the chain-carrying peroxyl radicals and on the stability of the phenoxyl radical formed in the reaction [17]. This has also been reported by many authors, indicating a considerable role of high phenolics content in antioxidant activity [160,161], and due to their several hydroxyl groups, flavonoids have been shown to be highly effective scavengers of various free radicals [162,163]. However, according to some others, the antioxidant activity of an extract cannot be predicted on the basis of its total phenolic content, and does not necessarily correlate with high amounts of phenolics. That is why both phenolic content and antioxidant activity information must be discussed when evaluating the antioxidant potential of extracts [164].

Scavenging of free radicals and inhibition of lipoxygenase are important target in the treatment of a variety of inflammatory diseases. Inhibition rate of 5-lipoxygenase is used as an indicator of anti-inflammatory activity, resulting in the inhibition of prostaglandin and leukotriene synthesis [11]. The study on the CN of Turkish origin included the EO and its major component PUL, which were tested for antioxidant and anti-inflammatory activity against standard active substances. The oil demonstrated inhibition on lipoxygenase at IC_50_ = 69.6 µg·mL^−1^, whereas its main component PUL showed no effect at the same tested concentration. Neither the EO nor PUL displayed radical scavenging activity (>0.5 mg·mL^−1^). Major component PUL showed no inhibitory effect on lipoxygenase activity, but other components despite of their low concentration demonstrated an inhibitory effect on lipoxygenase activity in 1/29 ratio when compared to the standard substance [11]. Another study on the material with the same origin comprised the antioxidant activity carried out using the DPPH (2,2-diphenyl-1-picrylhydrazyl) method. The IC_50_ values of the methanol, ethanol and petroleum ether extracts were found to be 4.78 ± 0.2, 10.19 ± 0.1 and 63.5 ± 1.25 µg·mL^−1^, respectively. The amount of total phenol was examined using the Folin-Ciocaltaeu method and it was found that the ethanol extract had the highest amount (187.33 mg GA·gr^−1^). Using the Aluminium Chloride (AlCl_3_) Colorimetric Method, it was determined that the methanol extract contained the highest concentration of flavonoid (21.7 mg catachol·gr^−1^) [148]. Investigating protective role of EO of Turkish CN against the oxidative stress of aflatoxin B_1_ in vitro, the oxidative status was assessed by measuring following oxidative stress markers: super oxide dismutase (SOD), glutathione peroxidase (GPx) and malondialdehyde (MDA). Major components of this oil were *cis*-piperitone epoxide, piperitenone oxide, limonene and terpinen-4-ol. It was observed that EO suppressed the mutagenic effects of aflatoxin B_1_ (AFB1) and modulated the adverse effects of aflatoxin B_1_ (AFB1). The results have clearly shown that the oil has strong antioxidative effect probably related to its action on the enzymatic activation system, and resulting from the role of *cis*-piperitone epoxide, piperitenone oxide and limonene compounds [60].

In vitro antioxidant activity of the ethanol extracts obtained from 21 aromatic plants from Greece belonging to the Lamiaceae family was investigated. Among them, that of CN exhibited the same activity as *α*-tocopherol [82].

Croatian material was assessed by DPPH scavenging activity and total antioxidant capacity assays, in comparison with hydroxycinnamic acids and trolox [165]. The total antioxidant capacity was 650.65 mg TE·g^−1^. Different extracts of Croatian origin have also been analyzed by four methods: DDPH, ABTS (2,2-azinobis (3-ethyl-benzothiazoline-6-sulfonic acid), reducing power and Oxygen Radical Absorption Capacity (ORAC) [17]. All examined extracts showed potent antioxidant activity reducing different radicals. Waste water after hydrodistillation revealed the highest activity. According to the authors, it was probably due the high content of phenolic compounds determined in this sample.

This result is in agreement with that found for two studied calamint species which showed different polyphenolic content and sterol composition [54]. The study on *C. grandiflora* indicated twice the polyphenolic content of CN, while the latter contained a higher number of sterols. Among them, stigmast-5-en-3β-ol was found to be the major constituent, and the methanolic extract of *C. grandiflora* was more potent than CN in a DPPH assay, while the activity of the *C. grandiflora* EtOAc fraction was weaker than its CN counterpart. Fractions of CN showed higher activity using a β-carotene bleaching test, and the petrol ether fraction of *C. grandiflora* showed significant inhibition of NO production. It is known that typical phenolics that possess antioxidant activity are phenolic acids that are found in this sample.

However, a weak antioxidant activity (EC_50_ = 410.7 µL) was detected for Italian plant material: neither the oil nor PUL, its main compound, displayed radical scavenging activity (more than 0.5 mg·mL^−1^). According to the authors, the EO of this species seems to have no environmental role [24]. CN hydroalcoholic extracts were prepared with ultrasound-assisted maceration from aerial parts of the plant collected in Italy during its different ontogenic growth stage (January, April, July and October). In this way, the authors explored phenophase influence on its polyphenol content, but the potential chemopreventive efficacy of the investigated extracts in terms of their antioxidant, cytotoxic, cytoprotective and anti-inflammatory activities were also assessed through an extensive biological screening. Each sample was analyzed for its phenol contents through Liquid Chromatography-Diode Array Detector-Electrospray Ionization-tandem mass spectrometry (LC-DAD-ESI-MS/MS) techniques, highlighting CN as a rich source of polyphenol compounds. Acacetin and caffeic acid derivatives were the main constituents and the relative abundance of each identified metabolite seemed to be strongly collection time dependent. The evaluation of the antioxidant capacity of the investigated hydroalcoholic extracts was carried out performing different tests, and Relative Antioxidant Capacity Index (RACI) was calculated as well. The extract from the summer collection exerted the highest antioxidant capability in cell-free systems, whereas that from winter collection was capable of exerting important cytoprotective and anti-inflammatory effects. Comparing phenol profiling data to bioactivity ones, it was highlighted that the winter extract contained an amount of acacetin and its derivatives nearly four times than those of caffeic acid derivatives [5].

Antioxidant activity of CN from Portugal was determined by three methods: DPPH, β-carotene/linoleic acid and reducing power assay, and the results obtained suggested the promising use of its EO and extracts as food supplement and pharmaceutical formulations [166].

The oil from Algerian material obtained by hydrodistillation was also evaluated by DPPH free radical-scavenging and reducing power. For both tests, the oil showed a low activity, which was less efficient than those of BHT and BHA. The authors explained it by its poor amount of phenolic components [97]. Another analysis also included the material from Algeria but the assessment in vitro of some extracts. The results showed that the aqueous extract exhibited good antioxidant activity with 90% inhibition rate at 4.62 mg·mL^−1^ by the DPPH method. The methanol extract showed the greatest capability in reducing power. However, this activity is lower in regard to ascorbic acid as a positive control [167]. The material of the same origin was also analyzed, but in terms of potential anti-inflammatory activity and the correlation of this effect with the plant’s potential antioxidant activity [53]. Methanolic extract exhibited the moderate inhibitory activity in the paw oedema induced by carrageenan (49% at 3 h), and had no effect on the TPA-induced ear oedema in mice. The antioxidant capacity of an extract largely depends on its composition and the conditions of the testing system used, and because many factors play a role, the effect of the extract cannot be wholly described with one single method. Total content in flavonoids and phenols of the extract was determined as 65.9 and 789 µg·mg^−1^, respectively. The scavenging properties of the extracts were measured in terms of their ability to bleach the stable DPPH^●^ and ABTS^●+^ radicals and galvinoxyl, with the activity values expressed as Trolox equivalents in the extract: 140, 537 and 313 µg·mg^−1^, respectively. The FRAP assay, which measures the antioxidant effect of a given substance in the reaction medium as reducing ability, was also performed. Activity values for this assay were expressed as ascorbic acid equivalents in the extract: 1227 µg·mg^−1^. The scavenger properties of the extracts against the superoxide anion and peroxynitrite were also determined: 6893 and 12.5 µg·mg^−1^. Finally, the lipid peroxidation of human plasma by the extract was evaluated with the aid of non-enzymatic generation systems. The extract was found to inhibit lipid peroxidation in human plasma, with inhibition value percentages greater than 80%.

The antioxidant activity of the samples collected in North Morocco has been tested by three most commonly used methods: DPPH, reducing power and β-carotene bleaching assay [95]. Compared to BHT, as a standard synthetic antioxidant, the oil showed relatively weak inhibiting activity in all the tests used. According to the authors, the poor activity of EOCN might be attributed to the lack of phenolic compounds, and to the high content of 1,8-cineole (42.94%), which has demonstrated poor ability of inhibiting oxidation [168].

Polyphenolic compounds, such as caffeic, chlorogenic and rosmarinic acids, have been identified from the aerial part of this plant [169]. Acacetin glycosides, together with linarin, have been reported as possible taxonomic markers in *Calamintha* genus [14]. The methanol extract from Egyptian material was found to contain flavonoids, phenolic acids, catechic tannins and mucilage, but also comprised traces of monoterpenes, mainly 1,8-cineole, carvone and PUL, from the glandular trichomes of the leaves. The flavones acacetin and linarin, as well as the flavanones eriodictyol and eriocitrine were also presented in the extract. Additionally, the extract exhibited significant DPPH free radical scavenging activity. It inhibited DPPH formation by 50% at a concentration of 68.57 mg·mL^−1^ (IC_50_) [170]. It is known that flavones, together with catechins, are among the most powerful flavonoids for protecting the body against reactive oxygen species and in the literature there are several reports that relate the biological activities of these compounds to their antioxidant effect [171].

Antioxidant activity of the plant extract from Iranian material was evaluated by Ferric Reducing Antioxidant Power (FRAP) and Total Phenolic Content (TPC) assays. The resulting TPC value was 0.75 ± 0.01 mg·g^−1^ indicating that the examined plant extract had significantly high content of phenolic compounds. The results also showed a considerable antioxidant activity and a high reducing power. In addition, a zymogram assay of peroxidase (POX) was performed to observe its activity. POX activity was observed as a brown color band and two separate bands were also observed, indicating at least two isoenzymes for POX [172].

## 8. Insecticidal Activity of EOCN

Plant insecticides have been used to fight pests for centuries. For instance, the use of plant extracts and powdered plant parts as insecticides was widespread during the Roman Empire. However, after the Second World War the few plants and plant extracts that had shown promising effects and were of widespread use were replaced by synthetic chemical insecticides. Later on, adverse effects of chemical insecticides became more evident with the appearance of problems like environmental contamination, residues in food and feed and pest resistance. Since the majority of plant insecticides are biodegradable, this has led to a revival of growing interest in the use of either plant extracts or EOs. More than 1500 plant species have been reported to have insecticidal value [40]. Many plant secondary metabolites, such as alkaloids, monoterpenoids or phenylpropanoids are toxic to insects; in addition, EOs extracted from plants have been widely investigated for pest control properties, with some demonstrating to be toxic [173].

Nineteen EOs from the island of Corsica (including CN) have been tested as potential repellents against mosquito *Aedes aegypti*. Space repellent properties were evaluated not highlighting the effect of CN as the promising one. Additionally, olfactory studies have been carried out on human volunteers showing great differences on a hedonic dimension and on the acceptance of these oils as fragrances for a repellent product. EOCN gave promising scores for both criteria. In addition to the repellent study and the olfactory tests, thermogravimetric analysis has been performed and CN was the most stable, with 61% weight loss at 33 °C over 24 h [174].

The Montenegran EOCN rich in monoterpene alcohol carvacrol showed strong insecticidal and fumigant activities against *Tribolium castaneum* adults [88]. Also, mortality rate of adult insects was tested: 56.67% of the insects died after 24 h, 83.33% after 48 h, while after 96 h the oil showed greatest toxicity and caused the death of 96.67%. Carvacrol has already been reported to have broad insecticidal and acaricidal activity against agriculture, stored products and medical pests, and it also acts as a fumigant [175,176,177,178]. Its insecticidal activity was also confirmed on the wine fly (*Drosophila melanogaster*), showing that carvacrol had a stronger activity than thymol, and that in combination with thymol, its insecticidal potential decreases. It could indicate that either other constituents are responsible for their toxicity, or that synergistic and/or antagonistic phenomena exist, and they alter the toxicity of the whole EO [179].

The potential toxic effects of three EOs (including EOCN) on adults and larvae of *Apis mellifera* were also studied. The LD_50_ of the EO on adult bees showed “virtually no-toxicity”. The in vivo assay of larval toxicity showed that CN was not toxic to larvae [180].

## 9. Phytotoxic Potential of CN

Terpenes play many ecological roles, such as the attraction of pollinating insects, the defense against herbivores and pathogens, and are also involved in plant-plant communication [181]. Their phytotoxic activity was widely demonstrated [182,183,184]. The individual and/or combined effects of the pure terpenoids found in CN methanolic extract and EOs were evaluated in in vitro bioassays, at different combinations and concentrations, on seed germination and seedling growth of *Arabidopsis thaliana* (L.) Heynh. To assess their potential phytotoxicity and their joint activity. None of the terpenes, singularly or in combination, was able to inhibit the germination process. Farnesene and *trans-*caryophyllene caused a strong inhibitory effect on root growth, and PUL, at the highest concentrations, caused a reduction on lateral root formation. Although the mixture of camphor−*trans*-caryophyllene, with or without farnesene, did not cause any effects on root growth, the addition of PUL induced a marked synergistic activity. Moreover, the addition of farnesene, at low concentration, to PUL−camphor−*trans*-caryophyllene mixture further increased the inhibitory effect on root elongation [185]. The results confirm that allelopathic effects are generally due to the interaction of a wide variety of molecules released by plants into the environment, and that the effects observed in nature cannot be always related to a single compound. Furthermore, the results suggest that the cooperative action among the compounds, in a natural community or in field, could reduce the threshold concentration needed to cause the phytotoxic effect, as observed with farnesene addition.

The phytotoxic activity of foliar volatiles of CN was assayed on germination and root growth of *Lactuca sativa* L. Moreover, the EOs extracted from the flowering plants were assayed on root growth of two crops, lettuce and radish (*Raphanus sativus* L.), and two of the most common weeds—*Lolium perenne* L. and *Amaranthus retroflexus* L. 

Foliar volatiles strongly inhibited both germination and root growth of lettuce, and its EOs, especially at the higher concentrations (125, 250 and 500 µL·L^−1^), inhibited both processes in lettuce, radish and *A. retroflexus* L. species while concurrently displaying little effect on *L. perenne* L. This effect appears to be important considering the potential use of EOCN or some of its constituents as a source of novel bioherbicides for weed management. Finally, the presence of bioactive terpenoids in leaf surface area and in EOs could suggest a potential role for this compound in CN establishment and proliferation in mediterranean habitats [77].

Another study included 17 wild plants from the Mediterranean area (also collected in Calabria) that were assayed for their allelopathic activity on *Lactuca sativa* L. and as a potential source of new natural herbicides for weed control. The aqueous extracts of shoots were assessed for their effects on seed germination and root growth of lettuce. Furthermore, to understand whether the most phytotoxic species could be a source of molecules for weed management, three different experiments were conducted to: (i) determine the persistence of their phytotoxicity during storage period; (ii) determine the phytotoxic potential of their decaying residues in pots against test species and (iii) evaluate their effects against on *Chenopodium album* L., *Sinapis alba* L. and *Echinochloa crus-galli* (L.) Beauv weeds.

The aqueous extract of CN drastically inhibited seed germination and root elongation of lettuce and weed species with *E. crus-galli* being the most tolerant one. The authors marked it as a promising source of bio-herbicides. Its allelopathic potential and the efficacy as a source of natural compounds were also confirmed in pot culture by adding the residues to soil mixture (simulating the field conditions) what resulted in reducing the shoot growth less than root growth of lettuce [186].

The bio-guided fractionation method was employed to isolate and identify some compounds, prerequisite for the possible future use of CN in weed management [187]. Leaves and stems were extracted with methanol and fractionated using *n*-hexane, chloroform, ethyl acetate and *n*-butanol, solvents with different polarity. The potential phytotoxicity of the methanolic extract and its fractions, evaluated by ED_50_ (effective dose) value comparison, were assayed in vitro on seed germination and root growth of lettuce. Germination and root growth of lettuce were strongly inhibited by catmint methanolic extract and its fractions, showing the following hierarchy of phytotoxicity for both physiological processes: ethyl acetate ≥ *n*-hexane > chloroform ≥ *n*-butanol. In the most active fraction, analyzed by HPLC, five polyphenols, gallic, vanillic, syringic, p-coumaric and ferulic acids, were identified and quantified. The *n*-hexane fraction was a mixture of 32 chemicals, mainly composed of terpenoids and fatty acids, as analyzed by gas chromatography-mass spectrometry (GC-MS). Further, GC analysis allowed quantification of five compounds: camphor, trans-caryophyllene, menthol, farnesene and pulegone. Furthermore, both fractions inhibited seed germination and root growth of two of the most common and noxious weeds in agriculture fields, *Amaranthus retroflexus* and *Echinochloa crus-galli* [188,189,190]. The results confirmed the phytotoxic activity of CN due to the presence of different molecule classes with biological activity and their potential future application as bio-herbicides [187].

The oil from the aerial parts collected in Italian Salerno during full blooming period was evaluated for its in vitro potential phytotoxic activity against germination and initial radical growth of *Raphanus sativus* L. and *Lepidium sativum* L., two species frequently utilized in biological assays, and three weed species *Sinapis arvensis* L., *Triticum durum* L. and *Phalaris canariensis* L. The oils seemed to be ineffective against germination and radical elongation of the five tested seeds. At the highest doses tested, the oil showed stimulatory activity of germination of garden cress (*L. sativum* L.) [24].

## 10. Anti-Ulcer Potential of CN

Gastric hyperacidity and ulcers are very common nowadays. It represents an imbalance between damaging factors within the lumen and protective mechanisms within the gastro duodenal mucosa [191]. The etiology of ulcer is not clear, and although prolonged anxiety, emotional stress, hemorrhagic surgical shock, burns and trauma are known to cause gastric irritation, the mechanism is very poorly understood [192]. Recently the involvement of neural mechanism in the regulation of stress responsiveness and complex neurotransmitter interactions were reported to cause gastric ulceration [193].

The treatment and prevention of these acid-related disorders are accomplished either by decreasing the level of gastric acidity or by enhancing mucosal protection [191]. In folk medicine, an infusion of CN leaves is employed to treat gastrointestinal diseases [61], and its eupeptic and carminative effects improve digestion [62]. Also, the herbs with high PUL content have been used as components of herbal teas for stomach disorders [194,195].

Determination of anti-ulcer potential of the material from India was undertaken [43]. Effect of various doses (0.4 and 0.8 mL·kg^−1^) of the oil was studied on gastric ulcers in pylorus ligation and Diclofenac sodium induced gastric mucosal injury in rats. Anti-ulcer activity was evaluated by measuring the ulcer index, gastric content, total acidity, and the pH of gastric fluid. It was noticed that the oil dose dependently decreased gastric content, total acidity, ulcer index and increased the pH of gastric fluid in pylorus ligation ulcer model. In Diclofenac sodium induced ulcer models, all oil doses decreased the ulcer index and increased the pH gastric fluid. These results supported the ethnomedical uses of oil of this plant in the treatment of gastric ulcer.

The gastroprotective activity of the methanolic extract was investigated using ethanol-induced ulcer in rats, with sucralfate as a reference drug. Samples of gastric mucosa, stained by PAS and haematoxylin/eosin, were observed by light microscopy. The significant results were obtained even after oral administration of the crude methanol extract of leaves, suggesting that this plant is able to preserve mucosal integrity against ethanol-induced gastric diseases. The efficacy of the extract was comparable to that of the reference drug. Moreover, oral treatment with gastroprotective doses of the extract did not affect either mice spontaneous locomotor activity or the behavioural and physiological functions. The authors concluded that the gastroprotective effect probably depended on a synergistic action of all the compounds occurring in leaves, even if the antioxidant potential of the leaves played an important role by removing damaging agents from the gastric mucosa [170]. The effect could be attributed, at least in part, to the presence of the flavonoids and polyphenols. The analysis of the methanol extract showed the presence of catechic tannins. Condensed tannins are able to protect the gastric mucosa by the inhibition of histidine decarboxylase and the resulting decreased synthesis of histamine [196]. The mucus accumulation, observed in the gastric mucosa of rats treated with the extract, could be related to a possible reaction between tannins and mucopolysaccharides [197]. Additionally, it is possible that the gastroprotective effect of the methanol extract was due, at least partly, to the presence of terpenes which were associated with antiulcerogenic activity in other plants [198,199]. After treatment with the extract, ethanol failed to damage the gastric mucosa of experimental rats. Light microscopy observations of the gastric mucosa of the treated rats showed an increase of the mucus layer, a normal straightness of the glands and a lower expansion of the glandular lumen and the vessels, compared with the control rats. Several pathways can be involved in this effect but the protective action of the methanol extract probably depends on an increase of mucosal barrier defense and the removal of damaging factors. The radical scavenger activity of polyphenols could be a more important mechanism, but the capacity of flavonoids to regulate the microcirculation may be another protective factor. Moreover, literature data report that flavonoids that have at least four hydroxyl groups and are of catechol-type in particular, as eriodictyol and its derivatives, can inhibit gastric H^+^, K^+^ ATPase, a proton pump that plays a pivotal role in acid secretion [200].

## 11. Additional Bioactivities of CN

The effect on the isolated rat ileum was also analyzed [28]. The material was collected in Montenegro and exerted significant spasmolytic effects which may underline the therapeutic action of the plant. The oil inhibited spontaneous contraction of the ileum, reaching its maximum effect at the concentration of 1 mg·mL^−1^ (EC_50_ value was 210.48 ± 9.12 μg·mL^−1^). The effect was reversible after washing, suggesting that the inhibition was not due to the damage of the intestine by the oil. In addition, PUL, the principal component in the oil, was analyzed as well.

Contractions of smooth muscles depend on Ca^2+^ influx from extracellular space through calcium channels, and it is well-known that the increase in external K^+^ concentration induces smooth muscle contractions through the activation of voltage operated calcium channels and subsequent calcium release from the sarcoplasmic reticulum [201,202]. The authors explained that the activity was probably caused by the inhibition of calcium influx through voltage-operated Ca^2+^ channels, which was confirmed by inhibition of K^+^ induced contractions with the oil (EC_50_ of 88.81 ± 6.01 μg·mL^−1^). The oil decreased the Ca^2+^ dose-response curves (EC_50_ of 18.18 ± 1.87 mmol·L^−1^), similar to that caused by verapamil (as the reference). PUL also exerted concentration dependent inhibition of spontaneous contraction of the ileum and the effect was 23 times as potent as the oil in inhibiting contractions. Accordingly, PUL may have a main role in spasmolytic activities of the plant [28]. 

Both the cytotoxicity and anti-cytotoxicity of the extracts from Turkey were evaluated on a non-cancerous cell line (L929) [203]. It was shown that water, *n*-BuOH and MeOH extracts have weak cytotoxic effects (500–1000 µg·mL^−1^) against cells tested. However, EtOAc and DCM fractions have significant cytotoxic effects with the IC_50_ values of 58.9 and 77.1 µg·mL^−1^, respectively. On the other hand, none of tested extracts recovered cytotoxic effect of 4-nitroquinoline 1-oxide (4NQO) at any concentration. The authors concluded that CN had no significant cell damage protective compounds but included some cytotoxic compound, recommending to be careful with their pharmacological usage.

Neutrophils are constitutively programmed to die by apoptosis, leading to phagocytic clearance of intact senescent cells by macrophages. For this reason, neutrophil elimination through apoptosis is of considerable interest as a mechanism for promoting the resolution of acute inflammation and avoiding a persistent inflammatory response. Moreover, reactive oxygen species produced by neutrophils, such as the superoxide anion, peroxynitrite anion and the hydroxyl radical, are responsible for tissue injury in many cases [204]. Potential anti-inflammatory activity of CN extract along with its apoptotic effect on the pro-inflammatory cells was analyzed [53]. As a result, no cytotoxic activity was detected at the selected concentrations.

The material from Turkey was assessed in terms of antigenotoxic activity against aflatoxin B_1_ [60]. It was evaluated by sister chromatid exchanges (SCEs) and micronucleus (MN) tests and it was observed that EO suppressed the mutagenic effects of aflatoxin B_1_. According to the authors, the activity can be attributed to the EO composition, and high *cis*-piperitone epoxide (48.66%), piperitenone oxide (22.08%) and limonene (13.51%) levels. Anti-genotoxicity mechanisms of terpenes are associated with their antioxidant capacity [205,206]. Therefore, in addition to the previous study, super oxide dismutase (SOD) and glutathione peroxidase (GPx) activities and malondialdehyde (MDA) levels were measured to determine the antioxidant effect. EO was observed to modulate the adverse effects of aflatoxin B_1_. The alfatoxin B_1_ treatment caused a decrease in SOD and GPx activities, but an increase of MDA level, while its effects on enzymes were decreased after treatment with the oil. The results clearly showed strong antioxidative and anti-genotoxic effects, which were probably related to the EO’s action on the enzymatic activation system, and resulting from the role of *cis*-piperitone epoxide, piperitenone oxide and limonene compounds [60].

The hypoglycemic effect in normal and streptozotocin-induced diabetic rats was also investigated [207]. The purpose of this study was to investigate the effects of a water extract from the aerial parts, after either a single dose or daily oral administration for 15 days on plasma blood glucose concentrations and basal insulin levels in normal and streptozotocin-induced (STZ) diabetic rats. The results clearly demonstrated the hypoglycemic effect of this plant extract in both normal and STZ diabetic rats. In addition, no changes were observed in basal plasma insulin concentrations after treatment with this plant in normal or STZ diabetic rats, indicating that the underlying mechanism of the plant's pharmacological action seems to be independent of insulin secretion.

The detailed analysis of different biological activities was done with Egyptian material [65]. The plant was found to present a very low toxicity both in vivo (LD_50_ of more than 100 mg·kg^−1^) and in vitro in the *Artemia salina* test (LD_50_ more than 500 µL·mL^−1^). The treatment with EO, in the Irwin test, showed a significant alteration in behavior, exhibiting typical effects of nonselective central nervous system (CNS)-depressant drugs; it potentiates the hypnotic effects of sodium pentobarbital, decreasing the induction time and enhancing the sleeping time, versus the control group treated with pentobarbital. Moreover, it produces a decrease in body temperature, which is not surprising as it is known that psychoactive CNS-depressant drugs reduce the body temperature [208]. The systemic administration of EO produced a protection against pentylenetetrazole-induced convulsions. The plant gave protection against generalized tonic-clonic seizures induced by PTZ, by increasing the latency period, by reducing the number of animals that exhibited convulsions, by diminishing the duration of convulsions, and by decreasing mortality to 67% at the dose of 100 mg·kg^−1^ [65].

Numerous aromatic plants are recognized as active in the CNS, and they have at least a hypothetical potential to affect chronic conditions, such as headaches, anxiety, depression, or epilepsy, that do not respond well to conventional treatments. Inhalation of EOs or their volatile terpenes has a significant role in controlling the CNS [209,210,211]. The oil compounds are lipophilic molecules, able to pass rapidly through the blood–brain barrier and to penetrate into the CNS, revealing a sedative effect [212,213]. According to some literature data [29,111], the components responsible for these activities appeared be the major monoterpenes: PUL, menthone and 1,8-cineole. Carvone reduces locomotor activity in the mouse, increases the latency period of PTZ-induced seizures, and potentiates pentobarbital-induced sleeping time [213,214]. The depressant effects of the oil analyzed could be related to its main component carvone, but it cannot be excluded that other constituents could act in synergy with carvone [65].

Investigation of antidiabetic activity of crude extracts and pure compounds from the aerial parts was undertaken [112]. The aqueous extract showed the promising antidiabetic activity. Based on this finding, it was fractionated giving two known hydroxycinnamic acids: rosamarinic and caffeic. Both acids exhibited significant activity, even more than the positive control, glibenclamide.

Antiproliferative activity of the plant extract from Iranian material was examined on human breast cancer cell line (MCF-7) using the 3-(4,5-dimethylthiazolyl)-2,5-diphenyl-tetrazolium bromide (MTT) method. The results showed a reasonable antiproliferative role played by the extract, indicating that this plant can be regarded as a suitable candidate for designing anticancer pharmaceutical preparations [172].

The ethanolic extract of CN growing wild in Croatia was tested for its inhibitory activity against AChE at concentrations of 0.25, 0.50 and 1 mg·mL^−1^ by in vitro Ellman’s method. The highest concentration demonstrated moderate inhibitory effect in a dose dependent manner with the IC_50_ value of 16.45 µg·mL^−1^ [165].

## 12. Pulegone—The Main EOCN Chemical Compound

(*R*)-5-Methyl-2-(1-methylethylidine) cyclohexanone is a monoterpene ketone, known as PUL, found mainly in the Lamiaceae oils. It was first isolated from the oil of *Mentha pulegium* L. (pennyroyal, *eng*.) from which its name is derived. It has a pleasant, refreshing odor which can be described as midway between peppermint and camphor [194].

**Scheme 1:**
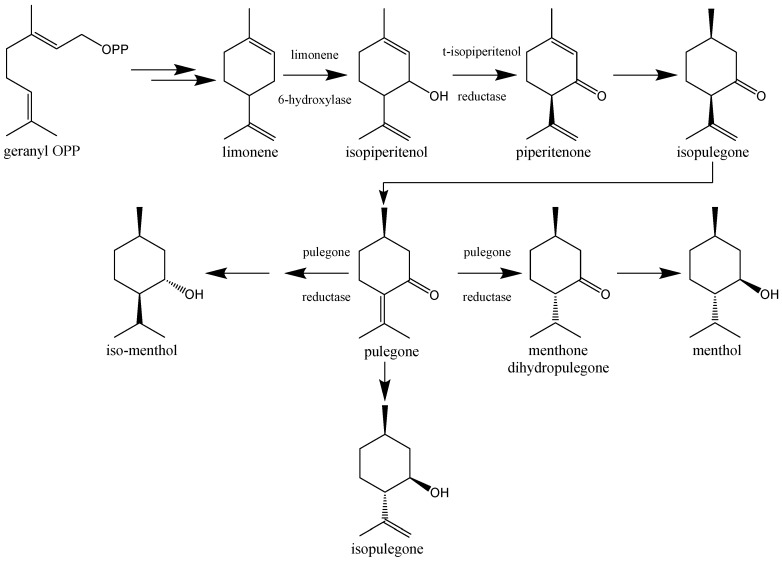
Biosynthetic pathway for PUL and its reduced forms [217].

PUL is practically insolubile in water but miscible with ethanol, diethyl ether and chloroform. Two enantiomers occur in nature, the R-(+)-form being the most abundant in the EOs [215,216]. Biogenetically, PUL is derived from terpinolene through piperitenone. It is also the precursor of menthone, isomenthone and isopulegone (Scheme 1). It is generally encountered in combination with one or more of the above mentioned compounds or its immediate precursor piperitenone [87].

This colourless oily liquid at room temperature with a strong pungent aromatic mint smell (C_10_H_16_O, molecular weight 152.23) has a density of 0.9346 g·cm^−3^. PUL has a vapor pressure of 138 mm Hg, a specific gravity of 0.937 at 25 °C. Its boiling point is 224 °C and it freezes at less than 25 °C. It is a flammable liquid (flash point of 82 °C) that will ignite if moderately heated. 

PUL is considered as one of the major common constituents of EOCN [11,33,34,42,44,48,51,55,57,70,77], where it can account for up to 85% [76]. Along with menthone, it represents the most frequent chemotype in the oils of this species, with clear difference in their ratio depending of plant’s phenophase stage. The initial process consists of PUL formation which is later (at the reproductive period) reduced in menthone. A slight difference in the relative ratio of PUL and menthone during and after the flowering stage of the plant was briefly reported [218], followed by more detailed studies [34,44]. The percentage of PUL was shown to be decreasing at the flowering period, while the content of menthone increased, leading to compositions in which the percentage of menthone was sometimes higher than that of PUL. This has been confirmed later in several studies [35,76].

This evolution of oil composition with the vegetative state seems to follow the one reported for *Mentha x piperita* L. [219]. The influence of seasonality on the PUL content in the EO of *Mentha pulegium* L. with a 54% reduction in its concentration during the winter has been also demonstrated [220]. PUL is the precursor for the formation of the stereoisomers of menthone, and this transformation leads to the reduction in PUL content of EOs. Variations in the compositions of EOs and PUL content depending on location, time of collection, and the stress to which the plant is exposed, among other factors, have also been explained [121]. Developmental and environmental factors are known to greatly influence the yield and composition of peppermint oil: the oil yield and menthol content increase with leaf (and thus oil gland) maturity, and a range of stress conditions (related to light, temperature and moisture status) tend to promote the accumulation of PUL and menthofuran [221].

PUL has been given Generally Recognized as Safe (GRAS) status by the United States Food and Drug administration since 1965. It is approved by Food and Drug Administration (FDA) for food use (21 CFR 172.515) and was included by the Council of Europe in 1974 in the list of artificial flavoring substances that may be added temporarily to foodstuffs without hazard to public health [194]. Therefore, it is widely used in flavouring agents, perfumery and aromatherapy. Limits in its use in food products have been issued for different applications, but there are currently no limits in the area of medicinal products. Its concentration in cosmetic formulations should not exceed 1% [222].

This oxygenated monoterpene exhibits a plenty of different biological activities such as antimicrobial [57,223,224,225,226,227,228,229], antihistaminic [230], antipyretic [29], convulsant [231,232,233], hepatotoxic [234,235,236,237,238,239] and hypercholesterolemic [240] among others. Also, it inhibits cytochrome P-450 [241] and lysozymal enzyme activities [242]. Inhibitory effect on the contractile activity of the isolated intestine [28,230] and myometrium [243] was demonstrated. It is a potent abortifacient [244]. PUL is reported to have anti-feedent, pesticidal and insect repellant properties [228,245,246,247,248]. Antiparasitical and anti-potato-sprouting activities of the plants with PUL-rich EOs have been reported [194,249]. Phytotoxic properties of PUL have been also analyzed [246,250]. Commercially, it is used as a flavoring agent for toothpastes and mouthwashes, and as a valuable ingredient for perfumes and various pharmaceuticals [228].

## 13. Alternative Sources of PUL

PUL is naturally found in plants of the Lamiaceae family, and its amount in various oils varies depending on several factors such as origin of the plant, yearly weather conditions, harvest date, plant age, fertilization, location, and planting time [215,251,252,253,254]. It is a major constituent of the volatile oils of European pennyroyal (*Mentha pulegium* L.) and American pennyroyal (*Hedeoma pulegioides* L.), where it comprises up to 97% and 82.3%, respectively [93,194,251,255,256,257,258,259,260,261,262,263]. High percentage has also been reported for some other mint species: 97.2% for *M. rotundifolia* (L.) Hudson [264], 86.2% for *M. gentilis* L. [265], 81.5% for *M. arvensis* L. [266], 78.4% for *M. requienii* Benth. [267] and 72.6% for *M. longifolia* (L.) Hudson [268,269]. It can be also found as a minor constituent in several other edible *Mentha* species and their derived volatile oils, including peppermint (*Mentha x piperita* L.) and spearmint (*Mentha spicata* L.) [239,270,271,272,273,274,275,276,277]. Other *Hedeoma* species can also be rich in PUL: 64.2% was found in *H. mandoniana* Wedd. [278] and 59.9% in *H. drummondii* Benth. [279].

Beside CN, as herein reported in detail, PUL can also be an important constituent in the EOs of some other *Calamintha* species. For instance, *C. sylvatica* Bromf has been found to contain from 11.6 to 54.5% of PUL [25,110,111], *C. grandiflora* (L.) Moench up to 35.2% [280,281], while the Turkish endemic *C. pamphylica* Boiss. et Heldr. contains up to 38% of PUL [194,282,283].

The oils rich in PUL are also obtained from other Lamiaceae species. For instance, 97% of PUL has been found in the oil of *Acinos majoranifolius* (Mill.) Šilić from Montenegro [284]. Up to 96.9% of PUL was reported for *A. suaveolens* (Sibt. et Smith) G. Don [285,286,287,288]. From this genus, *A. arvensis* (Lam.) Dandy (51.3%) [289] and *A. rotundifolius* Pers. (from 23.2% to 80.7%) [290,291] should be also mentioned. Some *Micromeria* species have been reported as a rich source of PUL: 80% was found in *M. capitellata* Benth. [292], up to 81% in different subspecies of *M. fruticosa* (L.) Druce [194,293] and 32.8% in *M. thymifolia* (Scop.) Fritsch [294]. The main EO constituent of *Satureja brownei* (SW.) Briq. from Venezuela [295] and *S. odora* (Gris.) Epl. from Argentina [296] was found out to be PUL (64.3% and 61.5%, respectively). Some other *Satureja* species also contain PUL: *S. glabella* (Michx.) Briq. (33.3%) and *S. darwinii* (Benth.) Briq. (11.4%) [297]. 

*Cyclotrichium niveum* (Boiss.) Manden. & Scheng. and *C. origanifolium* (Labill.) Manden. & Scheng. were found to contain up to 68% and 37% of PUL, respectively [23,298,299,300,301]. Up to 79.3% was reported for several *Minthostachys* species: *M. mollis* Griseb. [302,303], *M. andina* (Brett) Epling [277], *M. glabrescens* (Benth.) Epling [304] and *M. verticillata* (Griseb.) Epling [305].

The species from the genus *Ziziphora* are very rich in PUL content. 88% has been reported for *Z. brevicalyx* Juz. [306], 68% for *Z. hispanica* L. [307] and up to 65.2% for *Z. bungeana* Juz. [308,309]. A review [194] of all the analyses performed on Turkish *Ziziphora* taxa [50,310,311] pointed out a significant richness in PUL (up to 87%) in *Z. tenuior* L., *Z. taurica* Bieb. and *Z. clinopodioides* Lam.

Some *Hesperozygis* species (*H. marifolia* (Schauer) Epling, *H. ringens* (Benth.) Epling and *H. rhododon* Epling) have been found to contain up to 40.75% of PUL [228,312]. *Agastache* genus should be also mentioned, and some of its species have high amount of PUL: *A. rugosa* (Fisch. & Mey.) Kuntze, *A. scrophulariifolia* (Willd.) Kuntze and *A. mexicana* (Kunth) Lint & Epling contain 34%, 45.2% and 75.3%, respectively [313,314,315].

PUL-containing plant species have been also reported in some other families: Asteraceae [316,317,318,319,320], Cannabaceae [321], Apiaceae [322], Verbenaceae [323], Ericaceae [324], Rutaceae [325] and Myrtaceae [326]. Even some invertebrate animals (Bryozoan *Conopeum seurati*) have been reported to contain PUL [194,327].

**Scheme 2:**
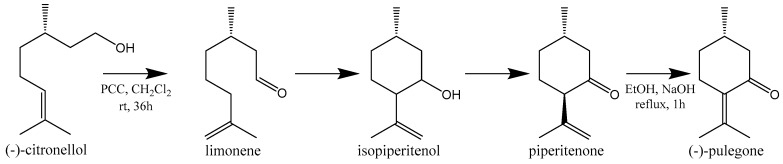
Cyclisation of (−)-citronellol to (−)-PUL.

Besides being available from natural sources, PUL can be also produced by chemical synthesis. A convenient synthesis of (−)-PUL from (−)-citronellol was demonstrated [328] (Scheme 2). The method included an ene cyclisation of (−)-citronellol under oxidative conditions: the treatment of (−)-citronellol with 2.5 equiv. of pyridininum chlorochromate (PCC) in dry methylene chloride gave *iso*-pulegone in one step via the intermediates citronellal and the *iso*-pulegols. Basic treatment of *iso*-pulegone with ethanolic sodium hydroxide gave (−)-PUL in 70% overall yield (Scheme 2). However, traditional PCC oxidation was found to be environmentally unfriendly and inconsistent results were obtained. Therefore, a mild organic oxidant (IBX) has been proposed to replace highly toxic PCC for the synthesis of chiral PUL [329]. The same method was reported by others [330], but the ene cyclisation of citronellal to *iso*-pulegol was achived by using catalytic ZnCl_2_. 

With another synthetic procedure (±)-PUL can be obtained from 3-methylcyclohexanone [331]. First, the oxo-ester obtained from 3-methylcyclohexanone via the glyoxilic ester was converted into the ketal, and by protecting the carbonyl group with a cyclic ketal, the β-oxo-ester can be simply converted into (±)-PUL.

It has been also reported that piperitenone could be enantioselectively hydrogenated to give (+)-PUL using chiral metal catalysts such as Rh complex with cyclohexylanisylmethylphosphine [332,333,334] or diphenylphosphine as the ligand [335], or Co complex with diphenylneomethylphosphine as the ligand [334]. Others have demonstrated the synthesis of (−)-PUL from simple cyclohexenone [335]; with the advantage of highly enantioselective conjugate addition of lithium cuprates to α,β-unsaturated ketone in the presence of pivaloylamidophosphine, (−)-PUL was obtained with 55% overall yield.

## 14. Toxicokinetic Studies on PUL and its Metabolic Pathways

Several toxicity studies of PUL and pennyroyal EO were performed. Acute lethal doses are available for PUL. The subcutaneous LD_50_ has been estimated as 1.709 mg·kg^−1^ in mice, the intraperitoneal LD_50_ as 150 mg·kg^−1^ in rats, and the intravenous LD_50_ as 330 mg·kg^−1^ in dogs [336]. Recently, the acute toxicity of the EO of *Mentha longifolia* (L.) Hudson in a rat model has been reported [337]. The noticeable signs of toxicity were observed when the oil was administered orally at doses greater than 100 mg·kg^−1^, with the LD_50_ value of 570 mg·kg^−1^. Abnormal gait, increased respiration, decreased activity and limb paralysis were found to be some of the clinical sings of PUL-mediated toxicity. Human ingestion of PUL (in pennyroyal oil) has been associated with toxic effects [338]. Moderate to severe toxicity from ingestion of at least 10 mL of pennyroyal oil was reported (coma, seizures and hepatic and renal effects as toxic symptoms), while less than 10 mL was generally associated with gastritis and mild central nervous system toxicity. However, the toxic effect was not always related strictly to dose and depended on the use of emetics or other treatments. For instance, coma and seizures associated with ingestion of 5 mL pennyroyal oil were also reported, as well as two other cases who survived after the ingestion of 30 mL [339]. This variability in the toxicity of pennyroyal oil may be due to its differing content of PUL [215].

EO of *Mentha pulegium* L. was suspected to be hepatotoxic, which was mainly related to PUL and its metabolites that are responsible for tissue necrosis [237,238]. Published animal toxicity studies have shown it to be primarily a hepatotoxicant and to a lesser extent a lung and kidney toxicant [234,235]. In humans, PUL (primarily from pennyroyal oil) has been associated with severe liver and kidney damage [338,340,341]. PUL is considered toxic due to a hepatic metabolic activation of the P-450 enzyme which liberates menthofuran [239]. According to others [244], it is metabolized to a series of hepatotoxins that causes liver cancer, and can rapidly destroy liver [236].

Toxicity studies in laboratory animals have mostly focused on its acute hepatotoxicity. However, pulmonary toxicity and cyst-like lesions in the brain have also been reported in mice and rats [235]. It was demonstrated that hepatotoxicity involved metabolism of PUL to menthofuran and further metabolism to a reactive γ-ketoenal [342]. The potential for oxidation of menthofuran to a reactive epoxide was also noticed [343,344], while other studies [345,346] detected potentially toxic *p*-cresol, a metabolite of menthofuran in the urine of PUL-treated rats. The covalent binding of ^14^C-PUL-derived radioactivity has also been observed, as an evidence for the formation of reactive intermediates, in the liver, kidney and lung of mice receiving 280 mg·kg^−1^ i.p. (intraperitoneal) [347]. PUL has been shown to deplete glutathione in plasma and liver of rats, and hepatotoxicity was increased if glutathione synthesis was inhibited with buthione-[*S,R*]-sulfoximine [236]. Mice receiving hepatotoxic doses of PUL had decreased glutathione levels within 3 h, followed by a rapid rise in plasma glutamic-pyruvic transferase [234]. The presence of glutathione conjugates in bile and mercapturic acids in urine was observed [348].

It has been noticed that i.p. administration of a single dose (250 mg·kg^−1^) of PUL to rats caused marked decrease in microsomal cytochrome P-450, aminopyrine-*N*-demethylase and glucose-6-phosphatase activities, as well as a significant increase in serum glutamate pyruvate transaminase (SGPT) level [349]. The authors also indicated the protective effect of phycocyanin, one of the major biliproteins of blue-green algae *Spirulina platensis*, on this kind of mediated hepatotoxicity. In order to find out the effect of phycocyanin on the mode of metabolism of PUL, experiments were carried out in vivo where urine samples collected from rats which where treated with PUL and rats which were treated with the combination of phycocyanin and PUL were analyzed. It was noticed that the level of menthofuran was significantly higher (nearly 70% more) in the urine samples collected from rats treated only with PUL. However, there were only marginal changes in the levels of other major metabolites. This is a significant observation since mentofuran is considered as the proximate toxin of PUL, responsible for at least half of the hepatocellular necrosis caused by PUL.

PUL was found not to be mutagenic in *Salmonella typhimurium* strains TA97, TA98, TA100, TA1535 or TA1537 at different concentrations (from 6.4 to 800 mg per plate) with or without S9 metabolic activation [350]. However, it was reported to be weakly genotoxic in *Drosophila melanogaster* at a dose of 0.2 mL, based on the presence of small single spots in the wing spot test [351], while the pennyroyal oil reported to contain 75.7% PUL was not mutagenic in the same assay at a dose of 2.1 mL.

PUL undergoes extensive hepatic metabolism with the primary metabolite being menthofuran [352]. In vivo and in vitro studies have shown that PUL is metabolized by the cytochrome P-450 enzyme system [347,353,354], while some authors [355] determined human CYP2E1, CYP1A2 and CYP2C19 to be active in metabolism of PUL to menthofuran. Several studies have been published describing the metabolism of PUL in rodents [342,345,346,356,357,358] indicating the complexity of the metabolic profile with at least three pathways involving hydroxylation, reduction or conjugation. One of the pathways leads to the formation of menthofuran involving 9-hydroxylation with a subsequent reduction of carbon-carbon double bond and furan ring formation. PUL can be reduced to menthone and isomenthone, followed by hydroxylation in ring or side chain and subsequent conjugation with glucuronic acid. The hydroxylation at C-5 or methyl (9- or 10-) to hydroxylated metabolites, followed by conjugation with glucuronic acid or glutathione can also take place. Then, formation of piperitenone after 5-hydroxylation is followed by dehydration; piperitenone is further metabolized by ring and side-chain hydroxylations (4-, 5-, 7-, 10-positions). Mercapturic acid pathway metabolites were detected in bile in mice and both bile and urine in rats [348]. The studies conducted at hepatotoxic doses (250 or 400 mg·kg^−1^) in male rats resulted in identification of 14 phase I metabolites, most arising from a common 9-hydroxy-PUL intermediate [346,356], while others [357] identified 10 phase II metabolites consisting of glucuronide, glutathione and glutathionyl glucuronide conjugates in the bile of Sprague-Dawley rats receiving 250 mg·kg^−1^ by i.p. injection. 14 urinary metabolites of PUL in rats, mostly glucuronide conjugates, have also been indentified [358]. In contrast to rats, a larger ratio of the ^14^C in female mouse urine consisted of specific glucuronide conjugates of the menthones. Mouse urine appeared to contain less of the glucuronide conjugate of 7α-hydroxymintlactone, derived from menthofuran. The authors did not report important sex differences in PUL metabolism.

Disposition of ^14^C-PUL after a single oral dose of 0.8–80 mg·kg^−1^ in male and female F344 rats and B6C3F1 mice have been investigated [348]. Mice excreted 85%–100% of the dose in 24 h, while rats excreted only 59%–81% of the administered radioactivity in the same time, primarily in urine (44% to 93% of total dose) and feces (6% to 24%), with a trace in expired air. Some dose-, species- and sex-dependent differences in elimination of PUL-derived ^14^C were also observed. For instance, the proportion of ^14^C excreted in the urine in rats decreased with increased dose, and male rats excreted less ^14^C than the female ones. Overall, mice excreted more ^14^C in urine than rats, and the amount of ^14^C excretion was similar (males) or higher (females) with increased dose. The study included the investigation of accumulation in tissues. PUL-derived radioactivity was present in liver, kidney, blood and lungs for 24 h following oral administration. Tissue concentrations were found to be lower in mice than in rats. The highest concentration of ^14^C in tissues of these animals was in the liver, a known target organ most closely associated with PUL toxicity [235]. Male rats tended to have higher tissue concentrations (even up to 10-fold greater) than females, especially in the kidney. This sex difference was not seen in mice. An additional study demonstrated binding of PUL and its metabolites menthofuran and menthone to α2u-globulin in the cytosol of male F344/N rat kidney following oral administration of these ^14^C-labeled chemicals [359]. Binding of the ^14^C to α2u-globulin was found to be reversible and did not result in accumulation of the protein in the kidney. Multidose studies in female F344/N rats indicated a potential for bioaccumulation of PUL-derived radioactivity in the liver [348].

Toxicology and carcinogenesis studies on PUL in F344/N rats and B6C3F1 mice have been reported recently [339]. They deposited PUL dissolved in corn oil through a tube directly into the stomach to groups of 50 male and female rats and mice for up to two years. Male rats received 18.75, 37.5 or 75 mg·kg^−1^ of PUL five times per week, while female rats and male and female mice received 37.5, 75 or 150 mg·kg^−1^ five days per week. Control animals received corn oil with no chemical added by the same method. As the result, there was no evidence of carcinogenic activity of PUL in male rats. Contrary to that, there was clear evidence of its carcinogenic activity in female rats based on increased incidences of urinary bladder neoplasms, as well as in mice based on increased incidences of hepatocellular neoplasms (adenomas in both sexes, hepatoblastomas in males). Rare bone lesions, osteoma and osteosarcoma, in female mice may have been also related to PUL administration. A unique kidney lesion, hyaline glomerulopathy, was observed in all dosed groups of mice (male and female) and in the groups of rats receiving the highest doses of PUL. The occurrence of some specifically localized nonneoplastic lesions (liver, nose, forestomach) was also associated with PUL administration.

Antitumor ativity of EO from *Agastache rugosa* (Fisch. & Mey) Kuntze as well as PUL, the main compound of this oil, using cell viability assay (MTT) have been evaluated [315]. The results showed a dose- and time-dependent increase of damage induced by all tested samples in gastric cancer cell line SGC-7901 with the inhibation rate of >85% for PUL at the concentrations ranging from 12.5 to 100 µg·mL^−^^1^.

## 15. Antimicrobial Activity of PUL

As a part of many EOs, PUL has been reported to play an important role in their antimicrobial activity. Antimicrobial activity of the EO of *Mentha x villosa* Hudson, as well as its major component rotundifolone and four similar analogues (including PUL) against standard strains of *S. aureu*s, *P. aeruginosa*, *E. coli, C. albicans* and one strain of meticilin-resistant *S. aureus* (MRSA) has been evaluated [227]. PUL was found to possess good antibacterial activity against the strains of *S. aureus* and MRSA (11 and 12 mm of inhibition zone, respectively), as well as antifungal activity against *C. albicans* (16 mm of inhibition zone), but no effect has been shown on the Gram(−) strains of *E. coli* and *P. aeruginosa*. According to others [360], PUL may be the most active antimicrobial component in *M. suaveolens* Ehrh. Strong antibacterial activity of *M. pulegium* L. has been attributed to the high amount of PUL [122,361]. The great synergism between its oil and mild heat has been related to the high content of PUL, especially at 4 °C [119,120,121,122].

Anti-dermatophyte activity against *Trichophyton rubrum* and *T. mentagrophytes* of the EO of *Minthostachys mollis* (Kunth) Griseb. was attributed to the presence of PUL as its main constituents [362]. In another study, this EO was found to be more active against Gram(+) bacteria, and the authors explained that to some extent by the presence of PUL (44.5%) [363].

PUL has been identified as a potent antimicrobial agent, particularly against all *Salmonella* species, showing inhibition halos varying ranging between 16 and 20 mm [57]. Its efficacy was maintained up to a dose of 0.5 mL, remaining appreciable at 1.0 mL with an inhibition halo of 10 mm. Strong antifungal activity of EOCN against *Fusarium* and *Aspergullus* strains has also been attributed to the high content of PUL [96]. PUL was found to strongly inhibite mycelial growth of *A. flavus*, and complete growth inhibition was observed at the concentration of 0.8 mg·mL^−1^ [229]. Other authors also pointed out the importance of PUL in antimicrobial effect of EOCN [55]. PUL, as the main constituent of the EO of *Micromeria thymifolia* (Scop.) Fritsch, was found to be responsible for its high antibacterial activity [226,294]. PUL isolated from *M. cilicica* Hausskn. ex P.H. Davis exhibited significant antibacterial and antifungal activities, particularly against *S. typhimurium*, *S. aureus* and *C. albicans* [146]. The antibacterial activity of *Ziziphora clinopodioides* Lam. EO may in part be associated with the presence of PUL as its main constituent [308].

## 16. Spasmolytic and Gastrointestinal-Related Activities of PUL

Many plant preparations are used as spasmolytics for the gastro-intestinal tract and may also be used for other disorders such as indigestion or diarrhea [364]. According to some authors [194,195] many of PUL-rich plants (many Lamiaceae species, including CN) are used as components of herbal teas for stomach disorders in Turkey. EOs such as those of peppermint, dill and caraway are examples of plant-derived spasmolytics [365]. It has been demonstrated that some monoterpenes also possess spasmolytic activity [366]. Investigating the correlation between structure and spasmolytic activity of rotundifolone and its analogues (including PUL) in ileum isolated from guinea-pig, the authors found that all monoterpenes tested were relaxants of intestinal smooth muscles. Comparison has been made for the effects of rotundifolone (having both α,β-unsaturated keto and epoxy groups) with PUL (having only a keto group) and limonene-oxide (having only an epoxy group), with the aim to find out possible influence of the presence of functional groups. As they concluded, both groups contribute to the spasmolytic activity of rotundifolone, but their presence is not a critical requirement. In order to investigate whether the position of the groups in the molecule affects the spasmolytic activity, rotundifolone was compared to pulegone oxide (the same positioned keto groups, but differ from each other in the position of epoxy group) and to carvone epoxide (with different positioned keto groups, but same epoxy group position). The results showed that the position of the functional groups at the ring also influenced the spasmolytic activity. In addition, the study showed the influence of chirality of the enantiomers on the pharmacological activity. Differently, the absence of the oxygenated molecular structure was not a critical requirement for the molecule to be bioactive.

Spasmolytic activity of PUL in guinea pig ileum has been investigated [367]. Beside its relaxant mechanism, the study also tested the involvement of voltage-dependent calcium and potassium channels and muscarinic antagonism. PUL caused a shift in the calcium curve to the right, with reduction in the maximum effect, and the pretreatment with tetraethylammonium chloride (TEA) partially inhibited relaxation produced by PUL. It also caused a shift in the bethanechol curve to the right, with reduction in the maximum effect. The results obtained showed that the intestinal muscle relaxation induced by PUL occurred via the partial blockade of Ca^2+^ channels, the activation of K^+^ channels and noncompetitive antagonism of muscarinic receptors. PUL (0.15–50 µmol·L^−1^) was also found to inhibit the spontaneous (EC_50_ of 9.02 ± 0.08 µg·mL^−1^) and K^+^ induced contractions of the isolated rat ileum (EC_50_ of 4.05 ± 0.14 µg·mL^−1^) and to run the dose response curve of calcium rightward. According to the authors, PUL may have the main role in spasmolytic activities of CN [28]. It exerted concentration dependent inhibition of spontaneous contraction of the ileum and the effect was 23 times as potent as EOCN in inhibiting contractions.

Modulation of the contraction of smooth muscle forms the therapeutic basis of several drugs, owing to the importance of smooth muscle function in most body organs, including airways, blood vessels, uterus, and gastrointestinal tract [368]. The importance of evaluating natural products that have biological activity on smooth muscle lies in the fact that spasmolytic substances are likely to have applications in the treatment of various diseases. This includes conditions such as cerebral vasospasm, asthma, hypertension, and uterine and intestinal spasms, as well as other pathophysiological processes that involve changes in the mechanisms of smooth muscle contraction and relaxation [369]. The vasorelaxant activity of PUL on the rat superior mesenteric artery was investigated [370]. Compared with other structural analogues, it was found that the absence of an oxygenated molecular structure was not a critical requirement for the molecule to be bioactive, but the position of ketone group in the *p*-menthone structure influenced the vasorelaxant potency and efficacy.

The antidiarrheal properties of the EO of *Mentha longifolia* (L.) Hudson in a rat model have been investigated [337]. The study revealed that EO possessed antidiarrheal activity, and according to the authors, the presence of PUL in the essence could be attributed to the antidiarrheal activity of the whole oil. This in vivo effects of the oil were consistent with the previous findings which indicated a reduction of total faeces weight and soft faeces frequency by PUL [371]. It was found that PUL was able to reduce the normal and altered propulsive movement induced by castor oil. PUL was also tested on gastrointestinal motility by charcoal meal test. An insignificant increase in the intestinal motility was observed in 100 mg·kg^−1^ of PUL, suggesting a weak spasmolytic activity. This activity was reverse dose dependent, with the greatest antispasmodic effect shown at 25 mg·kg^−1^ of PUL. It was more effective in the castor oil induced intestinal transit than in the normal transit, suggesting that PUL may be more effective in an altered state than in normal state.

The reduction in the intestinal motility may be responsible for the antidiarrheal activity. Probably, PUL at low doses increased the reabsorption of NaCl and water by decreasing intestinal motility as observed by the decrease in intestinal transit by charcoal meal and by their anticholinergic and antihistaminic effects [230]. In the enteropooling assay, the maximal effect of PUL was similar to loperamide, one of the most efficacious and widely used antidiarrheal drugs. Loperamide effectively antagonized diarrhea induced by castor oil [372]. The therapeutic effect of loperamide is believed to be due to its anti-motility and anti-secretory properties, and it is likely that PUL may mediate its effects through similar mechanisms. According to the results, PUL has weak anti-motility and very efficacious anti-secretory activity. Overall, these effects collectively may contribute to the appearance of no antidiarrheal activity. An antimicrobial activity of the PUL against common pathogens involved in gastroenteritis has been reported [373] and is likely to contribute to the antidiarrheal potency during infectious diarrhea.

## 17. Insecticidal Propertis of PUL 

Monoterpenoids have been considered as potential pest control agents because they are acutely toxic to insects and possess repellent [374] and antifeedant properties [375]. Their evaluation on various insects have established their biological activity as ovicides, fumigants or contact toxicants [376,377]. Acute toxicity of PUL to various insects was demonstrated [248], and according to some authors [351], it is the most powerful of three insecticides naturally occurring in many mint species. PUL has been considered effective as a defensive chemical, in part because of its repellency [245], but also because it interferes with insect feeding behavior, development, and reproduction [378]. Its use as a mosquito repellent has been pointed out [379]. Its acute toxicity was evaluated against the diamondback moth, *Plutella xylostella* (Lepidoptera), and the results showed moderate activity exhibiting less than 45% mortality in up to 15 µg/larva [380]. However, in biorational mixtures PUL was synergistic to both thymol and 1,8-cineole where the increase in activity was almost twofold. Notwithstanding their individual activities, linalool and PUL were also antagonistic in a mixture. Potential larvicidal activity against the western corn rootworm *Diabrotica virgifera virgifera* (Coleoptera) and adulticidal activity against the house fly *Musca domestica* (Diptera) have been investigated [246]. Among 34 naturally occurring monoterpenoids tested, PUL was one of the most effective with LD_50_ values of 39 µg per fly and 38 µg per rootworm. Pyrethrins were used as a standard, and in comparasion with PUL were almost five times more toxic. A soil bioassay for the determination of larvicidal activity of monoterpenoids in soil against western corn rootworm larvae was performed as well. PUL was found to possess moderate activity with LD_50_ value of 63 µg·g^−1^. In addition, the leaf-dip method was used to determine acaricidal activity of monoterpenoids against the twospotted spider mite *Tetranychtus urticae* (Acari: Trombidiformes). Mild activity was shown by 30 monoterpenoids tested, with toxicity differing depending on concentrations and exposure times. PUL caused 100% mortality at the highest concentration (10,000 pm) 24 h after treatment, but the lower concentrations showed no effect, or a slight effect was noticed after prolonged exposure time (10% mortality after 72 h for the concentration of 1000 pm). In another study [381], a preliminary fumigation screening test was evaluated on some important stored-product pest insects: the rice weevil *Sitophilus oryzae* (Coleoptera), the red flour beetle *Tribolium castaneum* (Coleoptera), the sawtoothed grain beetle *Oryzaephilus surinamensis* (Coleoptera), the house fly *Musca domestica* (Diptera) and the German cockroach *Blattella germanica* (Blattodea). Among twenty monoterpenoids tested, PUL was one of the highest activity causing 100% mortality in all five species tested at 50 µg·mL^−1^ air. In the fumigation assay, it was effective against *T. castaneum*; however the toxicity was relatively low in comparison to dichlorvos (used as a standard). The lowest LC_50_ value obtained for PUL was 0.2 µg·mL^−1^ at 37 °C and 96 h, while the corresponding treatment with dichlorvos had an LC_50_ value of 0.008 µg·mL^−1^. It was also found that the LC_50_ value tended to decrease at longer exposure times and higher temperatures, but to increase with the inclusion of a stored product: either maize kernels or house fly medium. According to the authors, it may be suitable as a fumigant or vapor-phase insecticide because of its high volatility, fumigation efficacy and safety. In addition, PUL was found to elicite the appropriate in vivo effects on *T. castaneum* paralysis and mortality [382]. Strong activity was also observed against *Frankliniella occidentalis* (Thysanoptera) and *Moechotypa diphysis* (Coleoptera) [383].

The fumigant effect of the oil of *Mentha pulegium* L. against adults of *S. oryzae* was also investigated [261]. The authors attributed it to the high content of PUL. The toxicity of this ketone against *S. oryzae* was also observed [384]. However, according to some authors [385,386,387], the insecticidal activity of the EO is not limited only to its major constituents and it could also be due to some minor constituents. Notably, it was confirmed that some “inactive” constituents may have synergistic effect on the “active” ones and that, although not active individually, their presence is necessary to achive full toxicity [388,389]. Some authors have even indicated better effectiveness of ketones compared with the structurally similar alcohols [377].

Larvicidal activity of PUL against the yellow fever mosquito *Aedes aegypti* (Diptera) was also investigated, showing high activity against all larval stages with LC_50_ values from 10.3 to 48.7 mg·L^−1^ [247]. Beside the acute toxicity, the authors also tested the possible synergistic effects of piperonyl butoxide (PBO), a well-known synergist that has been widely used to enhance the efficacy of natural or synthetic pyrethroids. Its addition significantly increased the activity of PUL. Additionaly, the ability of PUL to modify the ovipositional activity of *Ae. aegypti* was analyzed, and the strong repellent/deterrent activity was observed.

## 18. Other Bioactivities of PUL

The phytotoxic properties of PUL have been also analyzed. It has been characterized as one of the best candidates for chemical modification for the production of potential soybean (*Glycine max* (L.) Merr.) herbicides [250]. The study included the inhibition analysis of crop and weed seed germination, as well as seedling growth, by various monoterpenes. PUL was found to be among the inhibitoriest compounds to the greatest number of species, since it completely suppressed germination of five of the nine species tested, including three of four tested weeds. Ideally, a herbicide should completely inhibit germination and growth of target weed species, while having little or no effect on the crop. Accordingly, PUL had no effect on the soybean germination and inhibited just 46% of its seedling growth, while the weeds tested (*Amaranthus retroflexus* L., *Abutilon theophrasti* Medik., *Lolium multiflorum* Lam. and *Digitaria sanguinalis* (L.) Scop.) were completely or substantially affected by its addition.

A Styrofoam cup soil bioassay was used to evaluate phytotoxicity of monoterpenoids on corn plants and roots [246]. The authors have characterized PUL as the safer monoterpenoid in the experiment, which did not show any phytotoxicity at any concentration used. On the other side, it has been noted that all plants produce secondary compounds that are phytotoxic to some degree, placing PUL among the more phytotoxic ones of the hundreds of known plant-derived monoterpenes [390].

The strong activity of PUL against the cotton/southern root-knot nematode *Meloidogyne incognita* (Heteroderidae) was found [389]. Larvae immersion in test solutions of PUL for 24 h at the concentration of 468 µg·mL^−1^ achieved 100% paralysis. The EC_50_ value was estimated at 150 µg·mL^−1^. The study included different EOs and their main terpene constituents, showing higher nematicidal activity of terpenes when tested individually. Accordingly, it could be explained by the antagonistic action when they are part of EO. Similar result and EC_50_ value were obtained in the analysis against *M. javanica* [249]. 

Anti-inflammatory activity of PUL through the inhibition of inflammatory mediators releasing has also been demonstrated [391]. It was found that this terpene blocked prostaglandin and other inflammatory mediator’s formation in diarrhea which may in part explain its antisecretory effect. Antipyretic activity in rats of *Calamintha sylvatica* Bromf. EO has been reported, pointing out the responsibility of PUL and other major monoterpenes in exerting this effect [29]. PUL has been demonstrated to have moderate antioxidant activity [392]. Its radical scavenging activity and inhibitory effect on lipoxygenase have been investigated [11]. However, no effect was shown. It is well-documented that PUL is a potent inhibitor of acetylcholinesterase activity among ketones [382,393].

The effect of PUL on the central nervous system has been evaluated via a variety of experimental behavioural models in mice [233]. It was shown that PUL caused a significant decrease in ambulation and an increase in pentobarbital-induced sleeping time in mice, indicating a central depressant effect at a dose of 200 mg·kg^−1^ after i.p. injection. In contrast, it has been shown that PUL promoted ambulation, a CNS-stimulant action, in imprinting control region (ICR) mice via the dopaminergic system [232]. Further, it has been found that PUL (300 mg·kg^−1^ i.p.) also significantly increased the latency of convulsions, as assessed by the pentylenetetrazole (PTZ) method, and had an effect similar to that of diazepam, a standard anticonvulsant drug [233]. The antinociceptive properties of this monoterpene were assessed using several pain models. Chemical nociception induced in the first and second phase of the subplantar formalin test was significantly inhibited by PUL and was not blocked by naloxone. At the concentrations from 31.3 to 125 mg·kg^−1^ i.p., PUL dose-dependently inhibited both phases of the formalin test in a manner similar to that of morphine. This pharmacological property was confirmed by the hot plate test, which specifically measures central thermal nociceptive responses, showing the increase of the reaction latency of the mice by PUL addition. The results suggest that PUL is a psychoactive compound and has the profile of an analgesic drug. This is in accordance with several other studies on different monoterpenes and their pharmacological properties as psychoactive drugs [212,214,394,395,396,397,398,399]. The EO of *Calamintha sylvatica* Bromf. has been found to exert sedating activity in rats, and the authors attributed it to PUL and other major monoterpenes [29].

## 19. Conclusions and Future Perspectives

In general, plants have provided a source of inspiration for novel drug compounds. The increased interest in alternative natural substances is driving the research community to find new uses and applications for these substances and has led to a considerable increase in the use of medicinal plants. The results of the cited studies indicate that CN and PUL, as the main compound of EOCN, show a wide range of biological activities.

According to the *Flora Europeae* [15], CN includes two subspecies: *nepeta* and *glandulosa*. Having on mind their quite unstable taxonomic statuses, as well as various taxonomic treatments of the entire genus *Calamintha* Mill. (its uncertain separation from some other Lamiaceae genera), data collection seems to be inexhaustible. The existing literature abounds in synonyms, and even bigger problem arises at the subspecies level. Furthermore, some plant material has not sometimes been characterized in terms of subspecies (determined only to the level of species), which makes it particularly hard to draw conclusions and to compare data. Their separation seems to be irrelevant, especially taking into account the possibility of incompleted or even incorrect determination of material. On the other side, the question of valid recognition of these two taxa has been the subject of several studies including morpho-anatomical and phytochemical points of view [34,44,46,47,48,70,71,72,73,74]. Examination of the diagnostic characters between this two subspecies showes that many of them overlap. In addition, the evidence from phytochemical studies resulted in the conclusion that the chemical composition is quite independent of the subspecies and that they both can produce the same volatiles with *p*-menthane skeleton oxygenated in C-3. Consequently, their nomenclatural situation has been thoroughly discussed, making it impossible to distinguish two taxa in CN complex. Based on that, we have indiscriminately collected the literature data of one or the other subspecies, as well as of the numerous synonyms. Plenty of data about CN chemical composition has been discovered. The described chemotypes are diverse, and at least three can be distinguished with some exceptions. However, the most abundant one consists of PUL as the major oil constituent associated with menthone and/or isomenthone, menthol and its isomers, with possible low amounts of piperitone, piperitenone and/or their oxides. Reviewing the data presented herein, it seems quite reasonable to treat both subspecies as the same taxa, particularly having in mind their phytochemical properties and widespread traditional uses that, for sure, do not include recognition at any rate lower than a species level.

The contribution of each ingredient to the overall activity of an EO is a complicated pattern of interactions. The biological properties of the essence from the aromatic plants can be the result of synergism or antagonism, and may be attributable both to their major and minor components. Thus, investigation of the main EO compounds alone seems questionable. However, PUL is usually the dominant constituent (sometimes more than 80%) in EOCN and was found to reflect quite well the biophysical and biological features of the whole oil. According to many authors, it seems to be responsible for a lot of bioactivities, although it is possible that its activity is slightly modulated by the other minor molecules, as has recently been reported [76].

Considering all the available data about different biological activities of CN and PUL, the great values of this plant and its main constituent can be pointed out. This, for sure, justifies its traditional use in many cultures, as well as the use of other PUL-rich plant species. On the other side, the toxicological studies of PUL and its metabolites point to the need for precaution in exposure to it, thereby limiting its use in food products.

Moderate to strong antibacterial and antifungal activities were found for CN and PUL. However, to the best of our knowledge, the data indicating their antiviral effects are missing. Thus, that field can be considered as an interesting one for the future examinations. It should be added that further studies are needed to evaluate the in vivo potential in animal experimental models since there is little data on that aspect.

The studies on the antioxidant potential of CN have shown its weak to strong activity, which may be due to the different phenolic contents, possibly dependent of the sample origin. Contrary to that, there is little data available for PUL. Insecticidal properties of EOCN and PUL were thoroughly evaluated, revealing their good potential as insecticides, anti-feedants, repellents or fumigants. Their phytotoxic activities were also demonstrated, indicating the potential use of the oil or some of its constituents as a source of novel bioherbicides for weed management.

In folk medicine, an infusion of CN leaves is employed to treat gastrointestinal diseases, and its eupeptic and carminative effects improve digestion. Moreover, the herbs with high PUL content have been used as components of herbal teas for stomach disorders. For these reasons, CN and PUL have been subjected to a plenty of analyses aiming to prove its benefits. The results indubitably support the ethnomedical uses of this plant.

Additional bioactivities of EOCN, as well as PUL, have been investigated. The results showed good potential in relation to spasmolytic, hypoglycemic and sedative activities, among others. There is also a significant number of studies on different CN extracts that can be continued avenues of study in the future. Also, future studies should further explore the possible beneficial synergistic properties of combining PUL or EOCN with other natural or synthetic compounds.

## Figures and Tables

**Table 1:** *Calamintha nepeta* (L.) Savi, its subspecies and the most common synonyms.

Species	Subspecies	Synonyms
*Calamintha nepeta* (L.) Savi	*nepeta* L.	*C. nepetoides* Jord., *C. vulgaris* Clairv., *C. thessala* Hausskn., *Satureja calamintha* subsp. *nepetoides* (Jord.) Br.-Bl., *Satureja nepeta* (L.) Scheele, *Clinopodium nepeta* (L.) Kuntze subsp. *nepeta*; *Melissa nepeta* L.
	*glandulosa* (Req.) Ball	*C. glandulosa* (Req.) Bentham, *C. officinalis* Moench, *Thymus glandulosus* Req., *Clinopodium nepeta* (L.) Kuntze subsp. *spruneri* (Boiss.) Bartolucci et Conti and *Satureja calamintha* subsp. *glandulosa* (Req.) Gams, subsp. *nepeta* sensu Briq., subsp. *subnuda* (Waldst. et Kit.) Gams

**Table 2:** Chemical structures, names, MWs and CAS numbers of some of the most common constituents in EO of *Calamintha nepeta* (L.) Savi (EOCN).

Chemical Structure	Chemical Name	MW	CAS Number
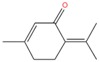	piperitenone	150.221	491-09-8
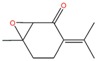	piperitenone oxide	166.220	35178-55-3
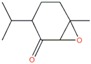	piperitone oxide	168.236	5286-38-4
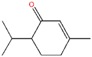	piperitone	152.237	89-81-6
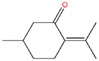	pulegone	152.237	89-82-7
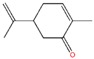	carvone	150.22	6485-40-1
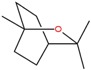	1,8-cineole	154.25	470-82-6
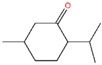	menthone	154.25	14073-97-3
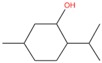	menthol	156.27	2216-51-5

**Table 3:** Chemical composition of groups 1–3 of the EOCN from Corsica expressed as relative percentage [34].

Constituents	IK^a^	IK^P^	Group 1		Group 2		Group 3	
			Mean	SD	Mean	SD	Mean	SD
α-Pinene	931	1022	3.2	0.5	1.0	0.6	0.7	0.3
Camphene	943	1066	0.1	0.1	0.1	0.1	0.1	0.1
Sabinene	964	1120	0.6	0.1	0.3	0.2	0.3	0.1
β-Pinene	971	1110	0.6	0.4	0.8	0.4	0.6	0.2
3-Octanol	977	1381	1.1	0.2	1.6	0.8	1.1	0.4
Myrcene	979	1159	0.5	0.3	0.9	0.4	0.5	0.2
Limonene	1020	1199	5.2	3.3	12.8	5.0	6.0	2.1
1,8-Cineole	1020	1209	0.5	0.4	0.7	0.5	0.4	0.2
*trans*-Sabinene hydrate	1054	1456	0.5	0.7	0.1	0.4	-	-
Linalool	1081	1544	0.5	0.3	0.9	0.5	0.3	0.2
Menthone	1135	1456	43.4	11.5	9.3	7.2	20.0	10.4
Isomenthone	1142	1481	3.2	4.3	0.4	0.6	2.0	1.9
Menthol	1156	1637	0.3	0.7	0.2	0.4	0.1	0.3
Neomenthol	1156	1594	1.6	2.8	0.7	0.8	0.4	1.1
Terpinen-4-ol	1161	1600	0.4	0.5	0.2	0.5	0.1	0.1
α-Terpineol	1172	1700	0.3	0.3	0.3	0.3	-	-
Dihydrocarveol	1178	1750	0.3	0.4	0.3	0.7	0.1	0.2
Pulegone	1216	1645	18.9	8.9	12.4	10.0	55.6	12.2
Piperitone oxide I	1230	1700	0.6	1.8	2.5	4.3	0.3	0.6
Piperitone oxide II	1230	1722	8.3	10.1	30.5	12.6	1.2	2.7
Piperitone	1232	1730	3.3	4.1	0.8	1.0	0.4	0.6
Thymol	1266	2189	-	-	0.2	0.2	-	-
Dihydrocarvyl acetate	1307	1657	1.8	2.4	2.1	1.7	1.2	1.3
Piperitenone	1315	1909	1.5	2.1	1.0	1.9	2.1	1.5
α-Terpinyl acetate	1332	1681	0.5	1.3	0.4	0.4	0.1	0.1
Piperitenone oxide	1333	1945	0.8	1.3	12.5	6.5	0.6	1.2
β-Caryophyllene	1419	1571	0.4	0.2	0.7	0.7	0.2	0.2
Germacrene D	1480	1704	0.4	0.2	0.9	0.8	0.4	0.3

IK^a^, IK^P^: retention indices on apolar and polar column, respectively. SD = standard deviation.
